# A scoping review of cloud computing in healthcare

**DOI:** 10.1186/s12911-015-0145-7

**Published:** 2015-03-19

**Authors:** Lena Griebel, Hans-Ulrich Prokosch, Felix Köpcke, Dennis Toddenroth, Jan Christoph, Ines Leb, Igor Engel, Martin Sedlmayr

**Affiliations:** Department of Medical Informatics, Friedrich-Alexander-University Erlangen-Nürnberg, Wetterkreuz 13, Erlangen, D-91058 Germany

**Keywords:** Cloud computing, Internet, E-health, Medicine, Healthcare

## Abstract

**Background:**

Cloud computing is a recent and fast growing area of development in healthcare. Ubiquitous, on-demand access to virtually endless resources in combination with a pay-per-use model allow for new ways of developing, delivering and using services. Cloud computing is often used in an “OMICS-context”, e.g. for computing in genomics, proteomics and molecular medicine, while other field of application still seem to be underrepresented. Thus, the objective of this scoping review was to identify the current state and hot topics in research on cloud computing in healthcare beyond this traditional domain.

**Methods:**

MEDLINE was searched in July 2013 and in December 2014 for publications containing the terms “cloud computing” and “cloud-based”. Each journal and conference article was categorized and summarized independently by two researchers who consolidated their findings.

**Results:**

102 publications have been analyzed and 6 main topics have been found: telemedicine/teleconsultation, medical imaging, public health and patient self-management, hospital management and information systems, therapy, and secondary use of data. Commonly used features are broad network access for sharing and accessing data and rapid elasticity to dynamically adapt to computing demands. Eight articles favor the pay-for-use characteristics of cloud-based services avoiding upfront investments. Nevertheless, while 22 articles present very general potentials of cloud computing in the medical domain and 66 articles describe conceptual or prototypic projects, only 14 articles report from successful implementations. Further, in many articles cloud computing is seen as an analogy to internet-/web-based data sharing and the characteristics of the particular cloud computing approach are unfortunately not really illustrated.

**Conclusions:**

Even though cloud computing in healthcare is of growing interest only few successful implementations yet exist and many papers just use the term “cloud” synonymously for “using virtual machines” or “web-based” with no described benefit of the cloud paradigm. The biggest threat to the adoption in the healthcare domain is caused by involving external cloud partners: many issues of data safety and security are still to be solved. Until then, cloud computing is favored more for singular, individual features such as elasticity, pay-per-use and broad network access, rather than as cloud paradigm on its own.

**Electronic supplementary material:**

The online version of this article (doi:10.1186/s12911-015-0145-7) contains supplementary material, which is available to authorized users.

## Background

Medicine is an increasingly data-intensive and collaborative endeavor [1]. Advances in the OMICS-fields (genomics, proteomics and the like) generate considerable amounts of data to be processed and stored. Secondary use of clinical data with text-or data mining algorithms also entails a growing demand for dynamic, scalable resources. Often these resources are only utilized temporarily so that permanent infrastructure investments are hard to justify and flexible on-demand services are sought alternatively.

Cloud computing seems a viable solution to fulfill these demands. Commercial providers like Amazon and Microsoft promise to make hundreds of virtual machines available at ones’ fingertips, almost immediately and just for the time they are really needed. The advantage of such offers is, that such resources only have to be paid for the configuration, size and time they are actually used.

Thus, the term “cloud computing” is described by the National Institutes of Standards and Technology (NIST) [2] as a model for enabling ubiquitous, convenient, on-demand access to a shared pool of configurable computing resources. As essential characteristics of cloud computing Mell and Grance have listed (1) on demand self-service, (2) broad network access, (3) resource pooling with other tenants, (4) rapid elasticity, and (5) measured services. Clouds promise advantages in dynamic resources like computing power or storage capacities, ubiquitous access to resources at anytime from any place, and high flexibility and scalability of resources. These benefits have been the reason for increasing adoption of cloud computing in many business areas. In recent years this concept has seemingly also been introduced in the healthcare domain. At least, a continuously increasing number of articles and publications appears in the popular literature and is provided by healthcare IT companies, but also in the scientific literature cloud computing for healthcare applications is gaining attention.

When reviewing the large amount of most recent literature dealing with cloud approaches in healthcare it becomes obvious, that many reports are dealing with cloud-computing technologies as a replacement for grid computing in the OMICS-field, while other fields of application (e.g. health information systems, health information exchange or image processing and management) still seem to be underrepresented. In the popular literature the application of cloud computing for healthcare information system provision for example is often used as a buzz word, but real evidence on research in healthcare cloud computing (beside the big topic of OMICS) or even its successful and resource saving application is missing. Researchers have proposed cloud computing as a new business paradigm for biomedical information sharing [3]. Kuo asked “if cloud computing can benefit health services” [4] and described opportunities and challenges of healthcare cloud computing [5]. Ahuja and colleagues have recently tried to survey the current state of cloud computing in the healthcare domain [6]. However, their overview has by far neither been representative nor comprehensive (many of their limited number of 27 references were company website information or publications with a commercial background).

Thus, since currently no real overview on the application of cloud computing in healthcare exists, it is the objective of our scoping literature review, to uncover the current myth on healthcare cloud computing. It is our aim to provide a comprehensive overview on the existing literature and elicit the key messages of the current publications. Further, we want to identify “hot spots” within the healthcare domain (but outside of the OMICS area) where cloud computing concepts and applications have mostly been discussed. For the articles published as “cloud computing application for health care” we wanted to check if the typical cloud computing service models (software, platform or infrastructure as a service) as well as their respective deployment models (private, community, public or hybrid cloud) are differentiated. Finally, we wanted to verify, how far the buzz word “cloud computing” has really already achieved more than only the “conceptual design” and “challenges” state and entered into the status of routine daily application, hopefully even with measures on its proven value for the healthcare domain.

Thus, our review questions were:

1. Does the existing literature provide enough evidence for the successful application of cloud computing in healthcare?
2. If the above question can be answered with yes:
  - What are the major application areas?
  - Are particular types of cloud concepts (public clouds, private clouds or hybrid clouds) more dominant than others?
  - Are particular cloud computing services (e.g. infrastructure as a service, software as a service, and platform as a service) more dominant than others?
  - Is there evidence, that the benefits, advantages and cost savings, which are typically assigned to cloud computing, could already be realized in healthcare environments?
3. If the above question must be answered with no:
  What are the barriers, which still need to be overcome in order to make cloud computing a successful technology also in the healthcare domain?

## Methods

Carrying out the review comprised the four stages of (1) collecting publications through a MEDLINE database search, (2) a first relevance screening to filter the results, (3) a review of the relevant papers and (4) a summarization of the content.

Within this review we consider the concept of healthcare to include all activities related to diagnosis, therapy and prevention of human diseases, or injuries, as well as clinical research and healthcare management. Publications on cloud computing for research in basic medical science (e.g. molecular medicine and genomics) however have not been considered.

### Search strategy

We searched the MEDLINE database in July 2013 and conducted an updated MEDLINE literature research in December 2014 for the terms “cloud computing” and “cloud-based”. Further, articles were subsequently included based on references in the publications of this first search.

All references were imported into the literature management program EndNote. All results were screened for relevance against our inclusion criteria.

### Selection of studies

The review team consisted of six researchers with expertise in medicine, computer science, medical informatics and statistics, working in groups of two. Each group was assigned one third of the papers in each round. Thus, each paper was reviewed independently by two reviewers. Conflicts between reviewers were resolved by short discussion rounds reaching a consensus.

At first, a relevance screening round based on the bibliographic data of a publication (type of publication, title, abstract, keywords) was conducted to remove obviously irrelevant papers. Details on this relevance screening are given in Additional file 1.

Excluded were papers on clouds in a non-computing sense (e.g. scatter plot analyses, clouds in a meteorological context) as well as cloud-computing in non-healthcare related topics (e.g. clouds used for biological analyses or for veterinary medicine). For the remaining papers, full-texts were obtained. If full-text was not available the article was excluded.

In the next step, based on the available full texts, papers published in languages other than English, editorials, letters to the editor, commentaries and press articles were excluded as non-scientific and out of the scope of this review. Articles dealing with cloud computing in genomics without a concrete relevance for patient care were excluded as they were not in the scope of our review. Additional file 2 provides the eligibility screening form used in this full-text screening step.

For the remaining papers, the content has been extracted as described in the following section.

### Full text screening and data extraction

The review protocol contained detailed instructions, inclusion/exclusion criteria, and a data extraction form (see Additional file 3). The data extraction form was handed to all reviewers in MS Office Excel 2010 format. This form included 14 closed and 8 open questions.

The closed questions captured e.g. the state of the described cloud computing system (i.e. theoretical, conceptual, prototype, successful), users addressed by the described system (e.g. physicians, patients, researchers), and the provider of the cloud (e.g. proprietary, i.e. self-constructed cloud computing solutions or commercially hosted solutions). Based upon NIST’s definition of cloud computing, its five essential characteristics (self-service, broad network access, resource pooling, rapid elasticity and measured service) [2] were checked for being mentioned by the authors. Besides advantages, also challenges were extracted, for example security concerns or dependencies on cloud providers.

Using open questions, the reviewers identified the main objective and the most important result of the article or of the described project, and in each case also summarized the specific usage of cloud computing. If mentioned, security concerns and countermeasures as well as cost considerations were noted. Finally, the definition of cloud computing, if it was used in a paper was collected.

Conducting this analysis of the articles enabled us to get an overview on the current state of research on cloud computing in healthcare and to collect the key messages of eligible publications.

## Results

### Record selection and article type

**Figure 1:**
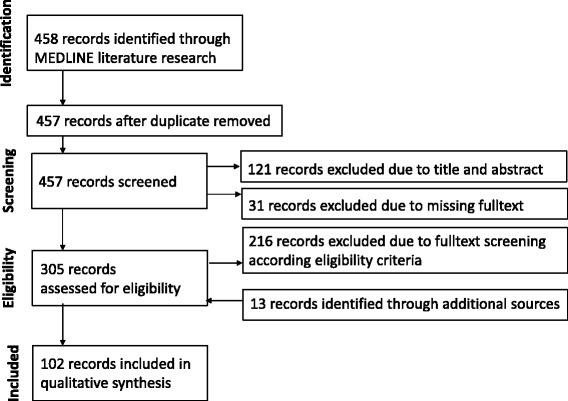
**Scoping literature review flowchart.**

Up to July 2013, 258 articles were found through literature research using the MEDLINE database. After the exclusion of one duplicate article and 63 articles, where title and abstract obviously illustrated that the contents of the article was from a completely different field, 194 remained for a cursory full text screening. Ten full texts were not available. 126 additional articles were removed during this step. 13 additional publications were identified from references of the screened literature, retrieved and included in the final analysis step. The first literature research thus resulted in 71 articles for the qualitative analysis. This literature research was updated in December 2014. During that research 200 further articles have been found in MEDLINE; 58 articles were removed due to their title and abstract. Of 21 articles the full text was not available; 90 further articles did not fit to the eligibility criteria. 31 new articles remained that were included in the qualitative analysis. Thus, in total 102 articles contributed to the subsequent qualitative synthesis. Of these 78 were journal papers, 24 were papers from conferences. The record selection process is shown in Figure 1. Additional file 4 gives an overview of all 102 articles that were used for the qualitative synthesis and includes detailed results of the characterization of all eligible reviewed articles.

**Figure 2:**
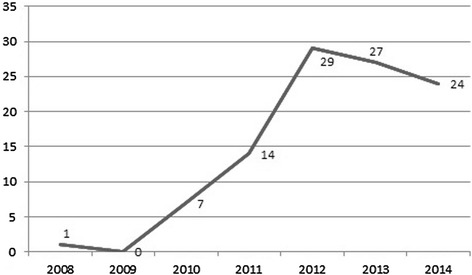
**Yearly distribution of published articles.**

Of the 102 articles only one has been published in the year 2008 and none in 2009. Seven articles have been published in 2010. From 2010 to 2011 the number of published articles concerning cloud computing in healthcare doubles up to 14 articles, and doubles again from 2011 to 2012 from 14 up to 29 articles. In 2013 27 articles have been published. Until December 2014 24 articles were identified–thus the trend seems not to be stable (Figure 2).

### Categorization of cloud computing research in healthcare: main domains

**Figure 3:**
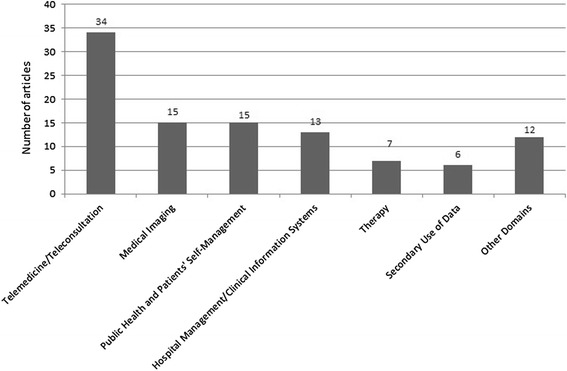
**Cloud computing in healthcare–main domains.**

The final list of papers was screened again to identify any new topic complementing the MEDLINE result list. Each two reviewers independently tagged the articles in the qualitative synthesis with main domains included in the papers. The final set of topics was discussed by all reviewers and similar topics were grouped to one main topic. Finally the following six domains for the application of cloud computing to healthcare, sorted in descending order by the number of included articles, were identified (Figure 3):

1. Telemedicine/Teleconsultation
2. Medical Imaging
3. Public health and patients’ self-management
4. Hospital management/clinical information systems
5. Therapy
6. Secondary use of data

Besides these categories we identified several articles that did not fit into one of the hot spots–these articles are explained in the “other domains” section.

In the following we describe the papers according to the identified categories, for more detailed information concerning MEDLINE articles’ content, please refer to the Additional file 4.

### Telemedicine/teleconsultation

Supporting communication and sharing data among stakeholders in healthcare is the most prominent domain including 34 articles. However, most publications describe just a typical telemedicine application when they report on the possibility to ubiquitously collect, access and share or analyze patient data from different hospitals or healthcare providers in dedicated health services networks.

Oshidori-Net2 for example is reported to be an “EPR and PACS sharing system” for six Japanese hospitals on an infrastructure which the authors call “server-based computing” and denote this to be cloud computing technology. The article further mentions that the server for this environment was built on virtual servers and virtual routers, but no further details on the cloud deployment or service model are given [7]. Similarly, Shih et al. [8,9] present a study in which 65 organ transplant healthcare professionals from China and Taiwan as well as 15 eHealth technology experts were questioned to identify pros and cons of so called “e-health documents” to be shared between institutions on a Web-platform. However, the article gives no information on why their proposal should be some type of cloud computing and not just a typical telemedicine platform for secure sharing of patient records for the respective organ transplant patients [8,9]. Rajkumar and Iyengar describe the concept of a Peer-to-Peer network to transfer medical resources like patient records and medical histories between diverse actors such as hospitals and ambulances in a medical emergency scenario [10]. In this scenario each hospital owns a community cloud to upload and share patient data with the nurse in the ambulance. A cloud application and an architecture test bed has been created, nevertheless the authors only present their concept and promise a reliable system to reduce the death rate in emergency care resulting from time delays during the patient transportation due to the missing opportunity to share important patient information with the hospital.

Also, Koufi et al. have named their concept “cloud emergency medical services” and provided a figure in which they depict their system components as infrastructure as a service, platform as a service and software as a service [11]. They further mention, that the system’s prototype implementation has been performed on a laboratory cloud computing infrastructure and that data are stored on multiple data centers in the cloud. Unfortunately, no further details on the specific type of cloud (private, community or public), the pay-for-use model or aspects of resource pooling with other tenants and rapid elasticity are given.

In another example Fujita et al. [12] called their implementation “Cloud Cardiology®”, mentioning, that “a cloud server enables to share ECG simultaneously inside and outside the hospital”. Nowhere in the further article itself, however, are any details presented why this server should really be a “cloud server” and not just a secured web-server for a telemedicine application, which provides a health information exchange platform in the internet.

Rao et al. propose a solution were also underserved, regions lacking infrastructure may benefit from cloud computing, without however illustrating in detail in which terms their approach should be a cloud application and not just a typical telemedicine service for rural areas [13]. Al-Zaiti et al. analyze the current problems and options for ECG transmission prior to hospitalization [14]. They see an option to standardize protocols used by different vendors and lower the investment cost for adopting the technology thanks to cloud services. One possible solution is presented by Fong and Chung [15] who describe a mobile cloud-based healthcare service by noncontact ECG monitoring. However, the software is described as client–server architecture implemented using standard web technologies and no cloud technologies are mentioned by the authors. Also Wang et al. [16] propose in their conceptual work a hybrid cloud computing environment to store data from personal health sensors worn at the body such as ECG sensors and to perform processing tasks. The purpose of the cloud is to accelerate computation intensive processing tasks by shifting them to the cloud server and therefore extend the battery life of mobile devices.

In contrast to the above examples, Hsieh and Hsu have presented a very comprehensive and detailed description of a 12-lead ECG telemedicine service based on cloud computing [17]. They have clearly described how the processing, visualization, management and e-learning services are deployed within the commercial Microsoft Azure cloud platform. They further present the reasons for adopting the Azure platform and the financial background of the implementation, based on the Azure pricing model with monthly costs directly related to CPU hours and GB storage used. As a second positive example we have identified the article of Hiden et al. who have described their development e-Science Central (a platform as a service which itself was built on an infrastructure as a service environment) [18]. Their article comprehensively illustrates not only the set of cloud services provided, which cover data storage services, but also service execution, workflow enactment and security. Finally as one of three case studies they present a medical pilot investigation (the MOVEeCloud project) where medical specialists assess the physical activity of patients based on data uploaded to the e-Science Central cloud by wearable accelerometers.

The improvement of the monitoring of discharged patients’ health-related quality of life and vital signs is the objective of caREMOTE, a prototype development of a cancer reporting and monitoring telemedicine system which is accessible by mobile devices [19]. For this prototype the cloud infrastructure was built on the Google App Engine (GAE) and data was stored in Google’s Big table technology. According to the authors, building such applications with GAEs sandbox technology leads to an isolation of the caREmote database within the cloud and secures the sensitive patient data from being violated. For a final routine application, this security aspect alone however, would by far not be sufficient. Therefore the authors intend to implement the Advanced Encryption Standard (AES) in a future version.

Similarily, Hussain et al. [20] implemented a system to use sensory data e.g. from smartphone sensors to detect activity patterns and ultimately lifestyle patterns. While the analysis was done on a local cluster of 4 host machines, the system is based on Hadoop as a typical big data technology which is easily scalable in clouds. Almashaqbeh el al. describe a cloud-based real-time remote health monitoring system (CHMS) which aims to integrate multi-hop sensor networks and cloud computing. However, the focus of the presentation is on routing the messages effectively (quality of service) through networked routers and computers and it therefore does not refer to any cloud or NIST characteristics [21].

The paper by Zao et al. puts a focus on telemonitoring in the neuroscience field [22]. The researchers present a prototypic online EEG-BCI (Brain Computer Interface) system based on wireless EEG headsets and mobile phones to predict users’ (patients, healthy persons) cognitive states in dynamic real-life situations. Cloud servers deliver the power to conduct semantic searches to find data segments matching with certain personal, environmental, and event specification used as a basis for the cognitive state prediction model.

### Medical imaging

One of the second largest domains of use with 15 articles is medical imaging focusing on the storage, sharing and computation of images.

Kakadis [23] provides a more theoretical description of various aspects of cloud computing with a special focus on medical imaging. Computing intensive image processing, sharing/workflows and archiving are the three major application areas, security the major challenge. As a visionary paper it remains on a conceptual level and does not explicitly refer to implementations. Similarly, Gerard also motivates the utilization of cloud technologies in radiology in his extended outlook, if adequate service level agreements are in place to guarantee uptime and performance and security is granted [24].

A cloud-based Picture Archiving and Communication System (PACS) might enable the storage of medical images as “PACS-as-a-Service” [25] or even provide a highly flexible “radiology round-the-clock” [26]. Rostrom et al. [1] have built a proof-of-concept prototype to demonstrate that the secure exchange of images between a client and a DICOM server hosted in the Microsoft Azure cloud is possible. The development of a DICOM (Digital Imaging and Communications in Medicine) compliant bridge for easily sharing DICOM services across healthcare institutions supports the provision of medical imaging services across the different institutions [25,27]. Also an efficient transport of large image files between PACS and image analysis servers is under development [28]. Doukas [29] implemented an Android client to receive patient information and images from a central server that runs in an Amazon virtual machine and measured download times of images via 3G and WLAN. Besides the server being in the Internet, neither details on the particular cloud-features nor on data protection/safety are issues mentioned.

Especially computationally intensive tasks are predestined to be put in a cloud computing environment. Cloud computing with its ability to lease computing capacities can be a suitable solution due to its pay-for-use approach, its ubiquitous access to data and its elasticity [30]. Maratt [31] compared the accuracy and efficiency of templating as part of the preoperative planning for total hip arthroplasty between traditional printing and a digital SaaS. While the outcome confirms that digital templating is quite as good as traditional methods, the article does not focus on the cloud per se, but more on the medical outcome as prerequisite for the acceptance of digital service. Yoshida et al. [32] describe the implementation of a framework for distributed image processing and positively evaluated the performance gained by using more processing units. However, the evaluation used multi-core CPUs in a single machine and the transfer to cloud-environments is mentioned only as an additional conceptual possibility. Similarly, Qu et al. [33] evaluated five image texture analysis methods using a “CometCloud” called hybrid cloud-grid distribution framework. Despite the cloud features, the evaluation reported was performed on a local, grid-like cluster. In contrast, Meng et al. [34] implemented a cone-beam CT reconstruction algorithm using MapReduce and evaluated it on 10 to 200 Amazon cloud nodes experiencing a 1/n decrease of computing time.

Supporting research, Avila-Garcia [35] describes the objectives of a Microsoft-funded project to implement a virtual research environment to lower the barriers to cancer imaging. While the paper cites some grid frameworks and enlists some general features required by researchers, no explicit links to cloud technologies are given when describing the functions to be implemented.

### Public health and patients’ self-management

Public Health is concerned with prevention, health promotion or improvement for individual citizens and patients but also for large population groups (epidemiology). Identically to the domain of medical imaging 15 articles belong to this domain.

Several papers include the idea that cloud computing might be used to support citizens and patients in managing their health status. Botts et al. [36] describe a pilot study named HealthATM which is a cloud-based personal health infrastructure to provide individuals from underserved population groups (i.e. people without health insurance) with instant access to their health information. The authors see cloud computing as a way to provide broad access to health data to population groups but do not explain how this highly scalable cloud architecture was implemented in detail, because the main focus of the paper was on the acceptance and usability of a personal electronic health records system in underserved populations.

The work of Piette et al. focusses on underserved patient groups as well. In two papers they describe how they created systems to inform underserved patient groups suffering from diabetes [37] resp. hypertension [38] with automated telephone calls to enable an improved self-management of the diseases. Although the authors mention that they use cloud computing to provide the application they do not differentiate between clouds and the Internet in general.

In their conference poster, Takeuchi et al. present a prototypic cloud-based system to store personal health and lifestyle data using mobile devices. In a cloud infrastructure they claim to have implemented data-mining technologies to extract individually important information such as lifestyle patterns. Although other persons like dietitians should have the possibility to add comments into the system it is not explained how data access in the cloud will be managed [39].

Similarly, the work of He et al. as well focusses on enabling citizens to manage their own health. They see cloud computing as a “component as a service” to develop a private healthcare cloud which should provide early warning of diseases [40]. Siddiqui et al. describe the concept of a Telecare Medical Information System (TMIS) which includes different medical services for patients and medical professionals such as a remote monitoring of physiological signals. The user should connect to the TMIS by using his/her smartphone and thus the smartphone needs to be equipped with authentication possibilities to ensure data privacy and data security. The authors propose a three-factor authentication (3FA) based on a dynamic cloud computing environment to enable the remote user authentication [41]. Van Gorp and Comuzzi discuss the prototype of MyPHRMachines where a cloud is used to deploy health-related data and the application software to view and analyze it in a personal health record system. After uploading their medical data to MyPHRMachines, patients can access them again from remote virtual machines that contain the right software to visualize and analyze them without any need for conversion. The patients should be able to can share their remote virtual machine session with selected caregivers [42].

Other projects are focused on specific user groups, such as the paper from Xu et al. [43] who worked on creating an automated cloud-based stress disorder monitor screening enabling patients suffering from Post-Traumatic Stress Disorder (PTSD) to monitor their progress during the treatment. According to the authors the so-called TPM (Tele-PTSD Monitor) system should be accessible via Public Switched Telephone Networks or via the Internet; latter might be realized using Amazon Elastic Compute Cloud. More information on the detailed cloud approach is not given to the reader.

Likewise, Su and Chiang describe IAServ (Intelligent Aging-in-place Home care Web Services) which is an electronic platform to provide healthcare services for elderly people at home. The objective of the platform is to prevent institutionalization of the users. Although the authors present an interesting architecture approach including an agent environment and a knowledge proceeding layer and explicitly mention the use of cloud computing services several times it remains unclear where a cloud computing system is used in the architecture of IAServ [44].

The work of Tseng and Wu as well focusses on enabling a healthy lifestyle of elderly people. They describe the prototype of iFit, which is a platform for the promotion of physical fitness to elder people through game-like activities. A so-called expert cloud is used to provide expert fitness diagnoses through a web service by receiving physiological data from the user and returning the corresponding fitness level and giving fitness suggestions to the user [45].

On a population level, Jalali et al. identified cloud computing as a solution to work with data of large populations by conceptualizing the use of virtual private clouds for public health reporting [46]. Price et al. worked on reducing execution time for epidemic analyses by using cloud structures [47]. Eriksson et al. describe a cloud-based architecture for simulating pandemic influenza outbreaks [48]. Ahnn et al. furthermore provide a theoretical paper on a way to create a cloud-based mobile health platform with a focus on energy efficiency [49].

### Hospital management and clinical information systems

Another interesting field of cloud computing in healthcare described by 13 articles is the deployment of clinical information systems into clouds. Commercial HIS vendors (compare e.g. the CSC Health Cloud [3]) have started to propagate new managed HIS services for their customers and also offer infrastructure as a service on a monthly payment basis. According to Low and Chen the selection of such an outsourcing provider needs to be evaluated very well. They proposed a provider selection evaluation model based on the Fuzzy Delphi Method (FDM) and the Fuzzy Analytic Hierarchy Process (FAHP) and identified decision criteria such as system usefulness, ease of use and reliability, high service quality or professionalism of the outsourcing provider [50]. Yoo et al. have chosen a more conservative approach by establishing a private cloud within Seoul National University Bundang Hospital (Korea) based on virtualization technology, a virtual desktop infrastructure and 400 virtual machines, which supported easy and overall access to each of the hospital’s information systems from all devices throughout the hospital. For this implementation they performed a five year cost-benefit analysis and showed that their approach reached its break-even point in the fourth year of the investment [51].

Two publications [52,53] describe the environment of two Romanian hospital departments with two different clinical subsystems which are capable to exchange data between each other based on HL7 CDA. Even though the authors introduced their article with a general description of the different cloud deployment and service models, the remainder of the articles provides no evidence of cloud-use or requirement.

As the Malaysian government initiated a paradigm shift to use electronic hospital information and management systems (HIMS) cloud computing could be the method of choice to reduce the escalating costs of data storing and sharing according to Ratnam and Ramayah. Although the authors do not describe this cloud system in detail, they mention that a cloud platform using Microsoft Windows Azure was used as prototype architecture [54].

In China, Yao et al. [55] created a community cloud-based medical service delivery framework (CMSDF) to enable the exchange of resources between a large general hospital with its associated smaller healthcare institutions–so called Grassroot healthcare institutions being the smallest administrative level of medical institutions in China including for example community health service centers or rural clinics. In the prototype CMSDF a cloud-based Virtual Desktop Infrastructure is owned and managed by a large hospital which is able to share its medical software as SaaS with the Grassroot healthcare institutions. According to the author for the 34 cooperative sanatoriums that participated, 89.9% of investment and maintenance cost were saved because the smaller facilities had not to buy and host expensive software on their own.

Rodrigues et al. specifically address the risks of hosting electronic health records on cloud servers [56]. The authors conducted a review of papers about security and privacy issues which different cloud computing providers currently use for the development of their platforms. They emphasize that shifting health resources to cloud systems needs the consideration of several requirements regarding privacy and confidentiality of patient data and mention that an external company was needed to audit the cloud platform provider’s security mechanisms.

### Therapy

Seven papers describe applications for planning, managing or assessing therapeutic interventions.

Chang et al. [57] describe a website for access to information on drug compounds used in Traditional Chinese Medicine. In future, the iSMART portal shall provide genetic research features for drug research; however, until now only a webserver to the database exists publicly and no information on the cloud-specific development is given.

Dixon et al. describe a prototype of a clinical decision support system (CDS) that packages a patient’s data and sends it to a remote SaaS for analysis, i.e. rule application [58]; a comparison of the local assessments versus the remotely generated results are analyzed in [59]. While the service model for cloud computing seems fulfilled, no features of SaaS such as scalability or pay-per-use are mentioned. Another evaluation of a cloud-based decision support system for early recognition of sepsis is described by Amlad et al. [60]. An add-on to the Cerner EHR was used to continuously monitor patient to recognize possible outbreak of sepsis. While the system performed well, the added benefit of being cloud-based is not described.

A large part of the papers from this domain evaluate the performance gain when moving Monte Carlo simulations for radiation therapy planning into the cloud. Poole et al. [61] used the Amazon Cloud to simulate a clinical linear accelerator and experienced a 1/n reduction of computing time usage when up to 20 worker instances were instantiated. Similarly, Miras et al. [62] used Microsoft Azure with up to 64 virtual machines of different sizes to measure a speedup of up to 37x. A more complex calculation is performed by Na et al. [63] who uses Amazon cloud with up to 100 worker instances for a speedup of 10-14x. The difference in the speedup is caused by the ability to parallelize the algorithms and the overhead for worker management and data communication. Cost of routine use has been estimated by all three to be below or at par of an equivalent local hardware cluster. However, all three studies are limited as the use cases focused on the performance of the mathematical libraries outside real world applications.

Also the paper of Parsons et al. [64] includes a description of an Amazon cloud-based model for Monte Carlo simulation of radiation dose. They used a web application called VirtuaLinac to model radiation treatment components.

### Secondary use of data

This domain includes articles describing cloud computing utilization for enabling secondary use of clinical data; e.g. for data analysis, text mining, or clinical research. Six papers belong to this domain.

Regola and Chawla discuss possibilities to store and share research health data and data from electronic health records in a cloud structure to reach an HIPAA (Health Insurance Portability and Accountability Act) complying environment [65]. For them, cloud computing offers the advantage of providing researchers with large computing resources. Data security can be achieved by providing proprietary cloud solutions where researchers can create their own customized networks and virtual servers.

Similarly, Chard et al. describe an approach to enable cloud-based services which should offer high scalability and HIPAA-compliant data security. They propose a cloud-based Software-as-a-Service NLP prototype to enable the extraction, procession, management, and comparison of medical data from several hospitals. Nevertheless, it does not become clear how data security should be achieved as-at the moment-the data in this cloud is not anonymous yet, but shall be accessible only to the particular data provider [66]. Also a cloud-based NLP service is described by Christoph et al. [67], here the free text is deidentified before put into the cloud. While the project described uses a community cloud, the OpenNebula-based implementation is said to run also in private or public scenarios. The main benefits of using cloud computing is in lowering the cost for processing data (no upfront investment, pay per use) and the managed services which enables the use of complex, computing intensive services by data providers with small IT departments.

Shen et al. describe generic standards-based services that can be transferred as virtual machines to other hospitals so that clinical pathways can be learned from order sets documented in EHRs. They mention that data mining models and results might be shared between different hospitals over a cloud-based server. Nevertheless the authors equal cloud computing with the Internet in general [68].

In the last article of this domain Rea et al. claim that they created a prototypic system that enables a cloud-based architecture to mine and normalize data for interchanging between hospitals [69]. The authors nevertheless do not explain how they face possible security and safety concerns when putting sensitive health data into a cloud, e.g. does this prototype include a private or a public cloud?

### Other domains

The main topic of some papers could not be assigned to one of the other categories.

Doukas et al. describe an infrastructure for automated skin lesion classification to detect skin cancer in an early stage. This assessment system is based on mobile technologies used by patients–a cloud provides the essential data processing components for pattern recognition [70].

Shen et al. implemented a cloud bio-signal (e.g. electroencephalography, electrocardiograph) analysis system but it hard to identify where exactly the cloud component can be found in their system architecture [71]. Papakonstantinou et al. describe the prototype of a semantic wiki to support training in healthcare process management which allows cost savings, accelerated time to delivery, and offloaded maintenance [72].

Second Live as a virtual environment is mentioned in two publications. Garcia-Penalvo et al. describe an interesting training environment for ongoing and already skilled pharmacists in virtual worlds [73]. Their objective is that students and teachers get each an own avatar in the Second Life environment to practice and train laboratory work to assure a high education and work quality. In the authors’ conceptual paper cloud computing is thought to support the mechanisms of data recovery and analysis to proper evaluate the processes in Second Life. Also Stoicu-Tivadar et al. propose a medical education approach based on the Second Life environment. They describe an information system that provides training for medical students to treat patients using avatars. According to the authors cloud computing should be used to store data bases such as a medical guidelines database remotely but no further details on the use of clouds are given [74].

Medical students may profit from radiology cases provided for use on mobiles according to Balkman and Loehfelm [75]. They build a learning web-portal based on Googles App Engine which was perceived well, although the latency of bringing images to mobile devices is seen as a downside. In the end, a student must be evaluated by his performance. Ferenchick and Solomon have developed a mobile assessment tool (basically web based questionnaires) for observers to document proved student skills [76].

Another work that is not captured by the defined domains is dealing with mobile health applications that require data-intensive multimedia and security algorithms–the authors refer to the cloud-based provision as “Security as a Service” [77].

An interesting approach is the work of Nagata et al., who successfully implemented a cloud-based EHR for reducing adverse health consequences of the earthquake and nuclear disaster in Fukushima in 2011. To allow the emergency teams in Fukushima an efficient management and handling of patient data, access to EHRs for assessing patient data was provided in the form of software as service [78].

Furthermore, we found one article containing a short SWOT (strengths-weaknesses-opportunities-challenges) analysis of cloud computing in healthcare [4], and another dealing with implementation of strategic planning of organizations moving to a cloud [5]. Finally, two “overview articles” have been identified: one provided an overview on data privacy solutions in cloud computing [79] and secondly, the work of Ahuja et al. names several benefits and challenges of cloud computing [6]. Both such overview approaches however, are not performed systematically, but only include some major thoughts on cloud computing in healthcare, its advantages and disadvantages.

### Implementation status

Our literature research revealed 22 theoretical papers that did not describe a specific cloud project but provided more common information on cloud computing in healthcare [4-6,8,14,23,24,26,49-52,56,79-87]. 12 articles include descriptions of basic conceptual work for cloud projects, but included no creation of a real system [16,30,35,46,53,54,63,77,88-92]. If applications are described they are usually in a prototype status [1,7,10-13,15,17-22,28,29,31,33,34,39-45,47,48,55,58,59,61-70,72-75,93-101]. Successful implementations of cloud systems in healthcare were only described in 13 of the 104 articles [9,25,27,32,36-38,51,57,60,71,78,102].

**Figure 4:**
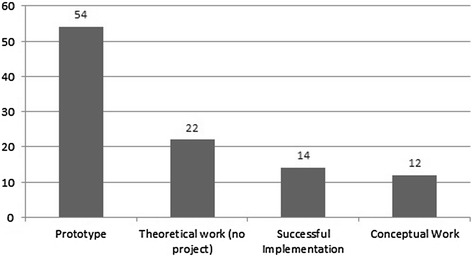
**Distribution of implementation status.**

The distribution of the diverse implementation status is shown in Figure 4.

### Definitions of cloud computing and NIST characteristics

Most articles rather describe features of the cloud than define it. These features include pay-as-you-go access to computing resources avoiding upfront investments and underutilizing private resources [1,21,25,30,31,53,62,65]. Scalability and flexibility are also presented as important characteristics for using cloud-based services, as system capabilities can easily adjust (scale) to momentary needs [1,25,50,62,82,84]. Availability and ubiquitous access are often mentioned [11,20,23,51,53,98] as well as the option to virtualize resources with distributed computing technologies, sometimes referred to as remote hosting [11,25,37,81].

These features can be linked to the five NIST characteristics of cloud-computing, i.e. rapid elasticity, followed by broad network access, resource pooling, on-demand self-service and measured service at least. But only eight publications directly cite NIST’s definition [4,5,18,23,24,40,52,55,63,67,75,81,83,85,100]. Thus, in most of the articles presenting cloud applications, details about the real deployment and service models remain unclear.

Some authors emphasize the more technical aspects of clouds: cloud computing is said to be more than just web-based applications, but also includes the necessary hardware, i.e. a physical network of many computers [5,26,77,84]. In five papers the cloud is even equated with the Internet in general [8,9,68,71,102].

### Users and providers

Most cloud-based services are provided using own, proprietary infrastructure. If commercial services are used, Amazon services are applied most often: Elastic Computing Cloud (EC2) is referred to eleven times [13,34,48,61,63,66,72,90,93,97] and Amazon’s S3 service (Simple Storage Service) three times [25,29,40]. One application [65] used the Virtual Private Cloud (Amazon VPC) to provide more secure services. Cloud infrastructures by other vendors such as Microsoft or Google play a minor role [1,12,17,19,36,62,75,98].

End users of the applications described are from five main groups: physicians, other medical staff, patients, clinical researchers, and IT experts. Several articles describe physicians and other medical staff storing, sharing and analyzing patient data or medical images [1,7-9,11-13,17,19,25,27-29,31,36,46,51,58,69,71,78,88,90,92,94,96,102]. Also therapy planning or simulation of radiation dose might be enabled for physicians using cloud systems [64].

Patients are mainly focused in projects on personal healthcare management [16,19,22,29,36-43,49,96]. Medical researchers should be enabled to access large pools of data for medical research purposes [18,30,35,57,65,66,68,69], whereas programmers and hospital IT staff should be enabled to work on the creation of cloud-based solutions [8,34,53,62,93,95].

### Challenges of cloud computing in healthcare

Three types of concerns using cloud computing in healthcare could be identified: safety/security of data as a threat to privacy, reliability and transparency of data handling by third parties, and lack of experience or evidence of a new technology.

First, in our literature we found that many authors mentioned data privacy and data confidentiality concerns. There is the fear that unauthorized persons might access sensible medical data in a cloud [1,5,13,18,24,25,28,29,31,46,51,52,63,65-67,72,81,83,85,88,94,96] which might hurt confidentiality of sensitive data about patients, therapies or physicians [41,103].

It is especially important that data security, privacy and confidentiality are focused [65] if handling of sensitive health data is outsourced to a commercial cloud, which means “that a third party now has control over the cloud-hosted area” [56]. Rodrigues et al. state that “cloud-based EHR must maintain the same level of data security as data stored in the servers of the health care provider” [56], but do not illustrate how this should be achieved.

In the US the Department of Health & Human Services has passed the Health Insurance Portability and Accountability Act (HIPAA) in 1996 which includes national standards for transactions in electronic healthcare concerning data privacy and security [104]. These standards provide a framework which should be considered when designing cloud services [1,63,65,66,83].

Many examples for improving data privacy and reducing confidentiality risks by authentication and authorization mechanisms are described [1,6,13,17,18,25,51,66,83,88,94]. For example, secure transmission protocols such as PCoIP could be employed, special security certificates could be utilized [66], access control lists (ACLs) can identify users’ role and the actions permitted [13,18], and licenses or electronic keys are handed over to authorized cloud users such as patients or physicians [88]. Further, a digital signature can ensure that data was entered or sent by the acclaimed person [56].

Data encryption is as well important to ensure data privacy [1,6,13,17,26,48,83,102]. Standardized encryption algorithms might be used [63,102] as well as secured data transmission using HTTPS [53,96]. Data encryption nevertheless can be problematic in emergency situations when physicians need instant access to patient data in a cloud and an access key is missing [58].

In principle, as few data as possible shall be put into the cloud [58]. Often it has to be anonymized before leaving an organization [30,65]. Sometimes it is possible to store identifiable data in separate entities to separate concerns [25]. Nevertheless, organizations often need to inform their patients before migrating their data to a third-party cloud computing provider [56]. The theoretical paper of Wang et al. focusses on the problem that cloud server providers might not be trustworthy (e.g. he might delete of modify some parts of the stored medical records). According to the authors an independent committee should be built to recover the original medical records from the cloud in the case of untrusted cloud providers [87].

Second, fears on technical issues exist when trying to implement a secure computing environment. Data might be lost due to technical problems with the cloud system [17,25,40,83] or vice versa, sensitive data cannot be fully deleted anymore once put into a remote cloud, leaving data in form of a fuzzy cloud structure [5,83]. In general, there is a fear of dependence on a cloud provider: a loss of control over their data [23,25,82]. Using audit trails might be a possibility to better control the use of a cloud system or facilitating data recovery [26]. This is why it is important to select only partners for outsourcing and cloud computing which can prove their security measures [56,89,100] and make handling of data completely transparent for the data owners [56]. Additionally, service-level agreements should be established between the customer organization and the cloud provider concerning data encryption and safety policies [23,100]. On the other hand clouds can even be utilized to store data using resource intensive security algorithms as kind of “Security-as-a-Service” [77].

Third, there are also concerns about the maturity of the cloud service–there might still be lack of evidence of successful cloud implementation in healthcare [4,83]. With regards to the economic advantages, Schweitzer proposes to conduct an economic analysis to ensure that savings through cloud computing are not overestimated because of hidden costs (e.g. cost for in-house IT support) [83].

## Discussion

Since for this relatively new domain with just emerging evidence standardized keywords and subject headings have yet not been well established, we decided on conducting a scoping review, as this approach is well suited for clarifying a complex concept and refine subsequent research inquiries [105]. While this approach yielded an overview on the status of cloud-computing in healthcare and identified the hot-topics, a systematic follow-up review could dig deeper into specific areas. Our review could help to focus on specific topics and to cope with the pace of publications.

The analysis of papers with regards to cloud features was hampered by the lack of information provided by the respective authors. Too often cloud was used synonymously with “Internet-based” or “running in a virtual machine” or “potentially scalable to a cloud” lacking any evidence of the real benefits. From the list of papers reviewed only eight papers refer explicitly to the NIST’s cloud computing definition itself. A large part of the papers do not even try to give a definition of cloud computing in general or describe in more detail what particularly makes their system to a cloud computing application. This is why we also conclude that future publications should more explicitly state their position with regards to the NIST characteristics.

Our findings may be limited by using MEDLINE as the main database as many publications especially in non-scientific media present cloud-based applications from a more practical or operational point of view. Searching the general Internet for cloud computing in healthcare reveals a very large number of hits of various kinds and qualities. Numerous cloud projects and offerings have not been scientifically published or evaluated. For example, CareCloud is a cloud-based software application including a complete infrastructure to document and facilitate caring processes in a hospital [106]; Box is a content sharing company which lately extends its cloud-based services to storage data to the healthcare sector enabling exchange of medical data between several physicians [107]. Well-known cloud services in health care are Microsoft’s Health Vault, a cloud-based platform to store and maintain health and fitness data [108] or the discontinued of Google Health service [109].

Of course, cloud computing is a hot topic and new papers are constantly published. Since 2010 the number of articles on cloud computing in healthcare has doubled almost every year. So the current review can only be a snapshot of a current state. However, comparing the publications date ranges of the topics shows no shift in the areas of research. A limitation is also that applications using cloud features may not be published with a title, abstract or keywords containing the word “cloud” and are thus not fitting our inclusion criteria.

## Conclusion

The aim of this review was to get an overview on the status of cloud-computing in healthcare and to identify areas of interest beyond typical “OMICS” topics. We found that especially resource intensive (e.g. medical imaging) and communication intensive areas such as various kinds of “tele-”applications are predestined for cloud computing use.

Considering our research objectives, we were able to a) provide a comprehensive overview on the existing literature and elicit the key messages of the current publications and b) identify the “hot spots” within the healthcare domain where cloud computing concepts and applications have mostly been discussed.

The question, if the buzz word “cloud computing” has really already achieved more than just the “conceptual design” and “challenges” state and entered into the status of routine daily application still needs to be negated. Only 14 of the 102 publications have described successful applications. The vast majority of papers still was in an early prototype stage or only described potential options, challenges and risks of cloud services for the healthcare domain, but no actual application.

Thus, even though from 2010 to 2012 the number of articles on cloud computing in healthcare has doubled every year we had to realize, that many publications do not reference the characteristics of cloud computing as defined by NIST [2]. A large part of the papers do not even try to give a definition of cloud computing in general or describe in more detail what particularly makes their system to a cloud computing application.

It appeared to us, that many researchers do already declare their application as a cloud computing application, if only the two features of broad network access for data sharing among different stakeholders and data access from everywhere are given. Such type of applications, however, have already been implemented for a long time and–as long as the scenario has focused on supporting patient diagnostics and therapy–such approaches are typically named telemedicine applications, health information exchange or personal electronic health records.

In our opinion, an application which really enhances its provision by means of cloud computing should explicitly describe the cloud-specific characteristics of their application following the NIST definitions, such as rapid elasticity or measured service where a pay-per-use model supersedes upfront investments. Resource pooling helps organizations to consolidate and simplify infrastructure services and continue existing trends in virtualization. While in the consumer market on demand self-services are often used, in healthcare environments they only seem to play a minor role. Authors should also illustrate how this new technology/business model makes their application more cost effective than without cloud technology.

Further, if cloud computing is a major feature of a healthcare application, we recommend that in future publications, authors do describe the particular deployment model chosen (which often also relates to a description of data privacy measures applied, being very important for sensitive personal health data) and also which particular type of cloud service is applied. In too many of the recent publications those descriptions were missing and the impression remained, that authors often called a typical internet-/web-based telemedicine application now a cloud application, just because cloud computing is a current buzzword.

## Additional files

Additional file 1: Table S1. Relevance screening form on basis of title and abstract. Shows how the large pool of MEDLINE articles found by keyword research was screened according to title and abstract.
Additional file 2: Table S2. Eligibility screening form on basis of full-text screening. Shows how the remained articles after the relevance screening where further screened on basis of eligibility criteria.
Additional file 3: Table S3. Characterization form on basis of full-text analysis. Shows how the content of the articles found eligible where characterized into several fields of interest.
Additional file 4: Table S4. Results from MEDLINE article analysis (n=102). Includes detailed results of the characterization of all eligible reviewed articles.

## Abbreviations

ACL: Access Control List
ATM: Automated Teller Machine
CDA: Clinical Document Architecture
CDS: Clinical Decision Support System
CPU: Central Processing Unit
CT: Computer Tomography
ECG: Electrocardiography
eHealth: electronic Health
EHR: Electronic Health Record
EPR: Electronic Patient Record
FAHP: Fuzzy Analytic Hierarchy Process
FDM: Fuzzy Delphi Method
HIPAA: Health Insurance Portability and Accountability Act
HIS: Health Information System
IaaS: Infrastructure as a Service
IT: Information Technology
NIST: National Institutes of Standards and Technology
NLP: Natural Language Processing
OMICS: genomics, proteomics, and the like
PACS: Picture Archiving and Communication System
PaaS: Platform as a Service
PHR: Personal Health Record
PTSD: Post-Traumatic Stress Disorder
SaaS: Software as a Service
SWOT: Strengths-Weaknesses-Opportunities-Threats analysis
