# Supplementary material for: A scoping review of cloud computing in healthcare
Source: BMC Med Inform Decis Mak. 2015 Mar 19;15:17. doi: 10.1186/s12911-015-0145-7 (PMC4372226; doi:10.1186/s12911-015-0145-7)
Supplement: Additional file 1: Table S1. — Relevance screening form on basis of title and abstract. Shows how the large pool of MEDLINE articles found by keyword research was screened according to title and abstract. [file 12911_2015_145_MOESM1_ESM.pdf]

Relevance screening form on basis of title and abstract

Question	Options	Exclusion if	Additional notes
**1. What type of source is the result?**	- Journal Paper (go to question 2)<br>- Conference Paper (go to question 2)<br>- Book Section (go to question 2)<br>- Unpublished Work (go to question 2)<br>- Commentary (go to question 2)<br>- Editorial (go to question 2)<br>- Others (e.g. press article)	- Result of category “others”	
**2. Is an abstract available?**	- Yes (go to question 3)<br>- No (go to question 2a)		- Please use existing links and try to find the abstract using search engines
**2a. Can it be concluded from the title that the article deals with cloud computing in healthcare?**	- Yes (go to question 3)<br>- No	- No	
**3. Does the article probably mean “cloud” in a computing context and not a homonym?**	- Yes (go to question 4)<br>- No	- No	- Other meanings for example cloud in a meteorological context
**4. Does the article deal with healthcare?**	- Yes (article will stay for further review process)<br>- No	- No	- Healthcare including: diagnosis, therapy, prevention of diseases, patient care, healthcare research, healthcare management
