# Supplementary material for: A scoping review of cloud computing in healthcare
Source: BMC Med Inform Decis Mak. 2015 Mar 19;15:17. doi: 10.1186/s12911-015-0145-7 (PMC4372226; doi:10.1186/s12911-015-0145-7)
Supplement: Additional file 2: Table S2. — Eligibility screening form on basis of full-text screening. Shows how the remained articles after the relevance screening where further screened on basis of eligibility criteria. [file 12911_2015_145_MOESM2_ESM.pdf]

Eligibility screening form on basis of full-text screening

Question	Options	Exclusion if	Additional notes
**1. Is the full-text available?**	- Yes (go to question 2)<br>- No	- No	- Please do the following to find full-text: Use Endnote/Citavi function to automate find full-texts; use available links; search university library's electronic journals; use search engine<br>- We also tried to find all full-texts via inter-library loan services<br>- Only articles where full-texts were not accessible after trying all of the above mentioned methods were excluded
**2. Is the full-text in English?**	- Yes (go to question 3)<br>- No	- No	
**3. Again: What type of source is the result?**	- Journal Paper (go to question 4)<br>- Conference Paper (go to question 4)<br>- Commentary<br>- Editorial<br>- Others (e.g. press article)	- Commentary<br>- Editorial<br>- Others	- We found that only by screening title and abstract the article type often is not identifiable<br>- From this step on only Journal Papers and Conference Papers are included
**4. Does the article**	- Yes<br>- No (go to	- Yes	- Leave articles which

	**mainly deal with cloud computing in "OMICS" and it does not have clinical relevance?**	question 5)		explicitly include a connection between genetic analyses and patient care<br>- Exclude article which deal with medical, genomic basic research; sequence analyses where patient care is just ONE of a large number of possible application area; chemical informatics or biological informatics papers; paper dealing with general methods to conduct genomic analyses
**5.**	**Is the article mainly dealing with cloud computing?**	- Yes (article will be kept for substantive analysis)<br>- No	- No	- Exclude articles which only refer to cloud computing as an example or an alternative solution; articles which only refer to cloud computing in the background chapter (e.g. "today CC enables the storing of big data. This is why a special imaging

	format is needed, this format is described in this paper”)
