# Supplementary material for: A scoping review of cloud computing in healthcare
Source: BMC Med Inform Decis Mak. 2015 Mar 19;15:17. doi: 10.1186/s12911-015-0145-7 (PMC4372226; doi:10.1186/s12911-015-0145-7)
Supplement: Additional file 3: Table S3. — Characterization form on basis of full-text analysis. Shows how the content of the articles found eligible where characterized into several fields of interest. [file 12911_2015_145_MOESM3_ESM.pdf]

Characterization form on basis of full-text analysis

Question	Options	Additional notes
1. **The article comes from which domain?**	- Medical Imaging<br>- Telehealth/Teleconsulting<br>- Public Health<br>- Therapy<br>- Hospital management/clinical information systems<br>- Secondary use of data<br>- Other domain [TEXTBOX]	
2. **Description of cloud computing use in the article**	[TEXTBOX]	
3. **Objective of the article/the project?**	[TEXTBOX]	
4. **Implementation status of the project?**	- Theoretical work (no project) (go to question 6)<br>- Conceptual work (go to question 6)<br>- Prototype (go to question 6)<br>- Successful implementation (go to question 6)<br>- Failed implementation (go to question 5)	
5. **Why did the implementation fail? (multiple answers possible)**	- Intended users did not adopt it<br>- Too expensive<br>- Too high efforts<br>- Technical problems<br>- Risks of cloud computing too high<br>- Other reasons [TEXTBOX]	
6. **Users of the cloud computing system? (multiple answers possible)**	- Patients<br>- Physicians<br>- Clinical researchers<br>- Hospital management<br>- Insurance companies<br>- Pharmacists<br>- Others [TEXTBOX]	
7. **Proprietary or commercially developed cloud computing system?**	- Proprietary development<br>- Commercial developement	
8. **Does the article name one specific cloud provider? (multiple answers possible)**	- Amazon<br>- Microsoft<br>- Google<br>- Others [TEXTBOX]	
9. **NIST characteristics:**	- On-demand self service<br>- Broad network access	- On-demand self-service:

	**Which are referred to in the article? (multiple answers possible)**	- Resource pooling<br>- Rapid elasticity<br>- Measured service<br>- None of these	The cloud provides computing capabilities, such as server time and network storage to the customer in an automated fashion. There is no need for human interaction with the service provider<br>- Broad network access: the cloud resource are available over the network and accessed through standard mechanisms<br>- Resource pooling: cloud computing is a multi-tenant model where multiple consumers are served with the provider’s computing resources. These resources

			include for example processing, storage, and memory.<br>- Rapid elasticity: the cloud resources can be flexibly provisioned according to customer demand<br>- Measured service: the usage of cloud resources can be monitored and controlled to provide transparency for the provider and the customer.
10.	**Which concerns do the authors have against cloud computing? (multiple answers possible)**	- Cost<br>- Security, safety, privacy concerns<br>- Dependence on cloud providers<br>- Others [TEXTBOX]	
11.	**Which security concerns do the authors have? (multiple answers possible)**	- Loss of data<br>- Violation of confidentiality and integrity of data<br>- Data cannot be deleted completely<br>- Violation of data protection laws<br>- Access to data by unauthorized persons<br>- Others [TEXTBOX]	
12.	**Could the authors minimize the risks?**	- Yes (go to question 13)<br>- No (go to question 14)<br>- Unclear (go to question 14)	
13.	**How did the authors minimize**	[TEXTBOX]	

**the risks?**	
**14. Do the authors think they could save money through cloud computing or do they fear to spend too much money?**	- Save money (go to question 15)<br>- Spend too much money (go to question 15)<br>- Unclear (go to question 16)
**15. How much money are they saving, spending in detail?**	[TEXTBOX]
**16. Definition of cloud computing in the article? Please cite**	[TEXTBOX]
**17. Important result of the article**	[TEXTBOX]
**18. Further important details that were not covered yet**	[TEXTBOX]
