# Supplementary material for: A scoping review of cloud computing in healthcare
Source: BMC Med Inform Decis Mak. 2015 Mar 19;15:17. doi: 10.1186/s12911-015-0145-7 (PMC4372226; doi:10.1186/s12911-015-0145-7)
Supplement: Additional file 4: Table S4. — Results from MEDLINE article analysis (n=102). Includes detailed results of the characterization of all eligible reviewed articles. [file 12911_2015_145_MOESM4_ESM.pdf]

Results from MEDLINE article analysis (n=102)

References used for qualitative analysis:

1. Abbas A, Khan SU. A review on the state-of-the-art privacy-preserving approaches in the e-health clouds. IEEE journal of biomedical and health informatics 2014;**18**(4):1431-41 doi: 10.1109/jbhi.2014.2300846[published Online First: Epub Date]|.

2. Ahnn JH, Potkonjak M. mHealthMon: toward energy-efficient and distributed mobile health monitoring using parallel offloading. Journal of medical systems 2013;**37**(5):9957 doi: 10.1007/s10916-013-9957-0[published Online First: Epub Date]|.

3. Ahuja SP, Mani S, Zambrano J. A Survey of the State of Cloud Computing in Healthcare. Network and Communication Technologies 2012;**1**(2):12-19 doi: 10.5539/nct.v1n2p12[published Online First: Epub Date]|.

4. Almashaqbeh G, Hayajneh T, Vasilakos AV, et al. QoS-aware health monitoring system using cloud-based WBANs. Journal of medical systems 2014;**38**(10):121 doi: 10.1007/s10916-014-0121-2[published Online First: Epub Date]|.

5. Al-Zaiti SS, Shusterman V, Carey MG. Novel technical solutions for wireless ECG transmission & analysis in the age of the internet cloud. Journal of electrocardiology 2013;**46**(6):540-5 doi: 10.1016/j.jelectrocard.2013.07.002[published Online First: Epub Date]|.

6. Amland RC, Hahn-Cover KE. Clinical Decision Support for Early Recognition of Sepsis. American journal of medical quality : the official journal of the American College of Medical Quality 2014 doi: 10.1177/1062860614557636[published Online First: Epub Date]|.

7. Lowering the Barriers to Cancer Imaging. eScience, 2008 eScience '08 IEEE Fourth International Conference on; 2008 7-12 Dec. 2008.

8. Bahga A, Madisetti VK. A cloud-based approach for interoperable electronic health records (EHRs). IEEE journal of biomedical and health informatics 2013;**17**(5):894-906 doi: 10.1093/bioinformatics/btu50510.1109/jbhi.2013.2257818[published Online First: Epub Date]|.

9. Balkman JD, Loehfelm TW. A cloud-based multimodality case file for mobile devices. Radiographics : a review publication of the Radiological Society of North America, Inc 2014;**34**(4):863-72 doi: 10.1148/rg.344130102[published Online First: Epub Date]|.

10. Botts NE, Horan TA, Thoms BP. HealthATM: personal health cyberinfrastructure for underserved populations. American journal of preventive medicine 2011;**40**(5 Suppl 2):S115-22 doi: 10.1016/j.amepre.2011.01.016[published Online First: Epub Date]|.

11. Chang KW, Tsai TY, Chen KC, et al. iSMART: an integrated cloud computing web server for traditional Chinese medicine for online virtual screening, de novo evolution and drug design. Journal of biomolecular structure & dynamics 2011;**29**(1):243-50

12. Chard K, Russell M, Lussier YA, et al. A cloud-based approach to medical NLP. AMIA Annual Symposium proceedings / AMIA Symposium AMIA Symposium 2011;**2011**:207-16

13. Chen TS, Liu CH, Chen TL, et al. Secure Dynamic access control scheme of PHR in cloud computing. Journal of medical systems 2012;**36**(6):4005-20 doi: 10.1007/s10916-012-9873-8[published Online First: Epub Date]|.

14. Chen YY, Lu JC, Jan JK. A secure EHR system based on hybrid clouds. Journal of medical systems 2012;**36**(5):3375-84 doi: 10.1007/s10916-012-9830-6[published Online First: Epub Date]|.

15. Cheng C, Stokes TH, Wang MD. caREMOTE: the design of a cancer reporting and monitoring telemedicine system for domestic care. Conference proceedings : Annual International Conference of the IEEE Engineering in Medicine and Biology Society IEEE Engineering in Medicine and Biology Society Conference 2011;**2011**:3168-71 doi: 10.1109/iembs.2011.6090863[published Online First: Epub Date]|.

16. Christoph J, Griebel L, Leb I, et al. Secure Secondary Use of Clinical Data with Cloud-based NLP Services. Towards a Highly Scalable Research Infrastructure. Methods of information in medicine 2014;**53**(6) doi: 10.3414/me13-01-0133[published Online First: Epub Date]|.

17. Cubo J, Nieto A, Pimentel E. A cloud-based Internet of Things platform for ambient assisted living. Sensors (Basel, Switzerland) 2014;**14**(8):14070-105 doi: 10.1016/j.jmr.2014.07.00210.3390/s140814070[published Online First: Epub Date]|.

18. Dixon BE, Simonaitis L, Goldberg HS, et al. A pilot study of distributed knowledge management and clinical decision support in the cloud. Artificial intelligence in medicine 2013 doi: 10.1016/j.artmed.2013.03.004[published Online First: Epub Date]|.

19. Dixon BE, Simonaitis L, Perkins SM, et al. Measuring agreement between decision support reminders: the cloud vs. the local expert. BMC medical informatics and decision making 2014;**14**:31 doi: 10.1186/1472-6947-14-31[published Online First: Epub Date]|.

20. Doukas C, Pliakas T, Maglogiannis I. Mobile healthcare information management utilizing Cloud Computing and Android OS. Conference proceedings : Annual International Conference of the IEEE Engineering in Medicine and Biology Society IEEE Engineering in Medicine and Biology Society Conference 2010;**2010**:1037-40 doi: 10.1109/iembs.2010.5628061[published Online First: Epub Date]|.

21. Doukas C, Stagkopoulos P, Kiranoudis CT, et al. Automated skin lesion assessment using mobile technologies and cloud platforms. Conference proceedings : Annual International Conference of the IEEE Engineering in Medicine and Biology Society IEEE Engineering in Medicine and Biology Society Conference 2012;**2012**:2444-7 doi: 10.1109/embc.2012.6346458[published Online First: Epub Date]|.

22. Eriksson H, Raciti M, Basile M, et al. A cloud-based simulation architecture for pandemic influenza simulation. AMIA Annual Symposium proceedings / AMIA Symposium AMIA Symposium 2011;**2011**:364-73

23. Fekr AR, Janidarmian M, Radecka K, et al. A medical cloud-based platform for respiration rate measurement and hierarchical classification of breath disorders. Sensors (Basel, Switzerland) 2014;**14**(6):11204-24 doi: 10.3390/s140611204[published Online First: Epub Date]|.

24. Ferenchick GS, Solomon D. Using cloud-based mobile technology for assessment of competencies among medical students. PeerJ 2013;**1**:e164 doi: 10.7717/peerj.164[published Online First: Epub Date]|.

25. Fernandez-Cardenosa G, de la Torre-Diez I, Lopez-Coronado M, et al. Analysis of cloud-based solutions on EHRs systems in different scenarios. Journal of medical systems 2012;**36**(6):3777-82 doi: 10.1007/s10916-012-9850-2[published Online First: Epub Date]|.

26. Fong EM, Chung WY. Mobile cloud-computing-based healthcare service by noncontact ECG monitoring. Sensors (Basel, Switzerland) 2013;**13**(12):16451-73 doi: 10.3390/s131216451[published Online First: Epub Date]|.

27. Fujita H, Uchimura Y, Waki K, et al. Development and clinical study of mobile 12-lead electrocardiography based on cloud computing for cardiac emergency. Studies in health technology and informatics. 2013/08/08 ed, 2013:1077.

28. Garcia-Penalvo FJ, Cruz-Benito J. Usalpharma: a cloud-based architecture to support quality assurance training processes in health area using virtual worlds. 2014;**2014**:659364 doi: 10.1155/2014/659364[published Online First: Epub Date]|.

29. Gerard P, Kapadia N, Chang PT, et al. Extended outlook: description, utilization, and daily applications of cloud technology in radiology. AJR American journal of roentgenology 2013;**201**(6):W809-11 doi: 10.2214/ajr.12.9673[published Online First: Epub Date]|.

30. Glasberg R, Hartmann M, Draheim M. Risks and crises for healthcare providers: the impact of cloud computing. 2014;**2014**:524659 doi: 10.1155/2014/524659[published Online First: Epub Date]|.

31. Haufe K. Proposal for a security management in cloud computing for health care. TheScientificWorldJournal 2014;**2014**:146970 doi: 10.1155/2014/26018710.1155/2014/146970[published Online First: Epub Date]|.

32. He C, Fan X, Li Y. Toward ubiquitous healthcare services with a novel efficient cloud platform. IEEE transactions on bio-medical engineering 2013;**60**(1):230-4 doi: 10.1109/tbme.2012.2222404[published Online First: Epub Date]|.

33. Hiden H, Woodman S, Watson P, et al. Developing cloud applications using the e-Science Central platform. Philosophical transactions Series A, Mathematical, physical, and engineering sciences 2013;**371**(1983):20120085 doi: 10.1098/rsta.2012.0085[published Online First: Epub Date]|.

34. Hussain S, Bang JH, Han M, et al. Behavior Life Style Analysis for Mobile Sensory Data in Cloud Computing through MapReduce. Sensors (Basel, Switzerland) 2014;**14**(11):22001-20 doi: 10.3390/s141122001[published Online First: Epub Date]|.

35. Hsieh JC, Hsu MW. A cloud computing based 12-lead ECG telemedicine service. BMC medical informatics and decision making 2012;**12**:77 doi: 10.1186/1472-6947-12-77[published Online First: Epub Date]|.

36. Jalali A, Olabode OA, Bell CM. Leveraging Cloud Computing to Address Public Health Disparities: An Analysis of the SPHPS. Online journal of public health informatics 2012;**4**(3) doi: 10.5210/ojphi.v4i3.4325[published Online First: Epub Date]|.

37. Ji Z, Ganchev I. A cloud-based X73 ubiquitous mobile healthcare system: design and implementation. 2014;**2014**:145803 doi: 10.1155/2014/145803[published Online First: Epub Date]|.

38. Kagadis GC, Kloukinas C, Moore K, et al. Cloud computing in medical imaging. Medical physics 2013;**40**(7):070901 doi: 10.1118/1.4811272[published Online First: Epub Date]|.

39. Kharat AT, Safvi A, Thind S, et al. Cloud Computing for radiologists. The Indian journal of radiology & imaging 2012;**22**(3):150-4 doi: 10.4103/0971-3026.107166[published Online First: Epub Date]|.

40. Klein CA. Cloudy confidentiality: clinical and legal implications of cloud computing in health care. The journal of the American Academy of Psychiatry and the Law 2011;**39**(4):571-8

41. Kondoh H, Teramoto K, Kawai T, et al. Development of the Regional EPR and PACS Sharing System on the Infrastructure of Cloud Computing Technology Controlled by Patient Identifier Cross Reference Manager. Studies in health technology and informatics. 2013/08/08 ed, 2013:1073.

42. Ubiquitous access to cloud emergency medical services. Information Technology and Applications in Biomedicine (ITAB), 2010 10th IEEE International Conference on; 2010 3-5 Nov. 2010.

43. Kuo AM. Opportunities and challenges of cloud computing to improve health care services. Journal of medical Internet research 2011;**13**(3):e67 doi: 10.2196/jmir.1867[published Online First: Epub Date]|.

44. Kuo MH, Kushniruk A, Borycki E. Can cloud computing benefit health services? - a SWOT analysis. Studies in health technology and informatics 2011;**169**:379-83

45. Lai CF, Chen M, Pan JS, et al. A collaborative computing framework of cloud network and WBSN applied to fall detection and 3-D motion reconstruction. IEEE journal of biomedical and health informatics 2014;**18**(2):457-66 doi: 10.1109/jbhi.2014.2298467[published Online First: Epub Date]|.

46. Langer SG. Challenges for data storage in medical imaging research. Journal of digital imaging 2011;**24**(2):203-7 doi: 10.1007/s10278-010-9311-8[published Online First: Epub Date]|.

47. Lenert L, Sundwall DN. Public health surveillance and meaningful use regulations: a crisis of opportunity. American journal of public health 2012;**102**(3):e1-7 doi: 10.2105/ajph.2011.300542[published Online First: Epub Date]|.

48. Low C, Hsueh Chen Y. Criteria for the evaluation of a cloud-based hospital information system outsourcing provider. Journal of medical systems 2012;**36**(6):3543-53 doi: 10.1007/s10916-012-9829-z[published Online First: Epub Date]|.

49. Lupse OS, Vida M, Stoicu-Tivadar L. Cloud computing technology applied in healthcare for developing large scale flexible solutions. Studies in health technology and informatics 2012;**174**:94-9

50. Maratt JD, Srinivasan RC, Dahl WJ, et al. Cloud-based preoperative planning for total hip arthroplasty: a study of accuracy, efficiency, and compliance. Orthopedics 2012;**35**(8):682-6 doi: 10.3928/01477447-20120725-05[published Online First: Epub Date]|.

51. Meng B, Pratx G, Xing L. Ultrafast and scalable cone-beam CT reconstruction using MapReduce in a cloud computing environment. Medical physics 2011;**38**(12):6603-9 doi: 10.1118/1.3660200[published Online First: Epub Date]|.

52. Miras H, Jimenez R, Miras C, et al. CloudMC: a cloud computing application for Monte Carlo simulation. Physics in medicine and biology 2013;**58**(8):N125-33 doi: 10.1088/0031-9155/58/8/n125[published Online First: Epub Date]|.

53. Mirza H, El-Masri S. National Electronic Medical Records integration on Cloud Computing System. Studies in health technology and informatics. 2013/08/08 ed, 2013:1219.

54. Morton T, Weeks A, House S, et al. Location and activity tracking with the cloud. Conference proceedings : Annual International Conference of the IEEE Engineering in Medicine and Biology Society IEEE Engineering in Medicine and Biology Society Conference 2012;**2012**:5846-9 doi: 10.1109/embc.2012.6347323[published Online First: Epub Date]|.

55. Na YH, Suh TS, Kapp DS, et al. Toward a web-based real-time radiation treatment planning system in a cloud computing environment. Physics in medicine and biology 2013;**58**(18):6525-40 doi: 10.1088/0031-9155/58/18/6525[published Online First: Epub Date]|.

56. Nagata T, Halamka J, Himeno S, et al. Using a Cloud-based Electronic Health Record During Disaster Response: A Case Study in Fukushima, March 2011. Prehospital and disaster medicine 2013:1-5 doi: 10.1017/s1049023x1300037x[published Online First: Epub Date]|.

57. Nkosi M, Mekuria F. Cloud computing for enhanced mobile health applications. 2nd International Conference on Cloud Computing Technology and Science (CloudCom). Indianapolis, USA: NY: IEEE, 2010.

58. Parsons D, Robar JL, Sawkey D. A Monte Carlo investigation of low-Z target image quality generated in a linear accelerator using Varian's VirtuaLinac. Medical physics 2014;**41**(2):021719 doi: 10.1118/1.4861818[published Online First: Epub Date]|.

59. Papakonstantinou D, Poulymenopoulou M, Malamateniou F, et al. A cloud-based semantic wiki for user training in healthcare process management. Studies in health technology and informatics 2011;**169**:93-7

60. Patel RP. Cloud computing and virtualization technology in radiology. Clinical radiology 2012;**67**(11):1095-100 doi: 10.1016/j.crad.2012.03.010[published Online First: Epub Date]|.

61. Piette JD, Datwani H, Gaudioso S, et al. Hypertension management using mobile technology and home blood pressure monitoring: results of a randomized trial in two low/middle-income countries. Telemedicine journal and e-health : the official journal of the American Telemedicine Association 2012;**18**(8):613-20 doi: 10.1089/tmj.2011.0271[published Online First: Epub Date]|.

62. Piette JD, Mendoza-Avelares MO, Ganser M, et al. A preliminary study of a cloud-computing model for chronic illness self-care support in an underdeveloped country. American journal of preventive medicine 2011;**40**(6):629-32 doi: 10.1016/j.amepre.2011.02.014[published Online First: Epub Date]|.

63. Poole CM, Cornelius I, Trapp JV, et al. Radiotherapy Monte Carlo simulation using cloud computing technology. Australasian physical & engineering sciences in medicine / supported by the Australasian College of Physical Scientists in Medicine and the Australasian Association of Physical Sciences in Medicine 2012;**35**(4):497-502 doi: 10.1007/s13246-012-0167-8[published Online First: Epub Date]|.

64. Poulymenopoulou M, Malamateniou F, Vassilacopoulos G. Emergency healthcare process automation using mobile computing and cloud services. Journal of medical systems 2012;**36**(5):3233-41 doi: 10.1007/s10916-011-9814-y[published Online First: Epub Date]|.

65. Price RC, Pettey W, Freeman T, et al. SaTScan on a Cloud: On-Demand Large Scale Spatial Analysis of Epidemics. Online journal of public health informatics 2010;**2**(1) doi: 10.5210/ojphi.v2i1.2910[published Online First: Epub Date]|.

66. Qi X, Kim H, Xing F, et al. The analysis of image feature robustness using cometcloud. Journal of pathology informatics 2012;**3**:33 doi: 10.4103/2153-3539.101782[published Online First: Epub Date]|.

67. Rajkumar R, Sriman Narayana Iyengar NC. Dynamic Integration of Mobile JXTA with Cloud Computing for Emergency Rural Public Health Care. Osong public health and research perspectives 2013;**4**(5):255-64 doi: 10.1016/j.phrp.2013.09.004[published Online First: Epub Date]|.

68. Dhatri - A Pervasive Cloud initiative for primary healthcare services. Intelligence in Next Generation Networks (ICIN), 2010 14th International Conference on; 2010 11-14 Oct. 2010.

69. Ratnam KA, Dominic PD, Ramayah T. A structural equation modeling approach for the adoption of cloud computing to enhance the Malaysian healthcare sector. Journal of medical systems 2014;**38**(8):82 doi: 10.1007/s10916-014-0082-5[published Online First: Epub Date]|.

70. Rea S, Pathak J, Savova G, et al. Building a robust, scalable and standards-driven infrastructure for secondary use of EHR data: The SHARPn project. Journal of Biomedical Informatics 2012(0) doi: 10.1016/j.jbi.2012.01.009[published Online First: Epub Date]|.

71. Regola N, Chawla NV. Storing and using health data in a virtual private cloud. Journal of medical Internet research 2013;**15**(3):e63 doi: 10.2196/jmir.2076[published Online First: Epub Date]|.

72. Ribeiro LS, Costa C, Oliveira JL. Enhancing the many-to-many relations across IHE document sharing communities. Studies in health technology and informatics 2012;**180**:641-5

73. Rodrigues JJ, de la Torre I, Fernández G, et al. Analysis of the Security and Privacy Requirements of Cloud-Based Electronic Health Records Systems. Journal of medical Internet research 2013;**15**(8):e186

74. Rolim CO, Koch FL, Becker Westphall C, et al. A cloud computing solution for patient's data collection in health care institutions. 2nd International Conference on eHealth, Telemedicine, and Social Medicine. New York: NY: IEEE, 2010.

75. Rostrom T, Teng CC. Secure communications for PACS in a cloud environment. Conference proceedings : Annual International Conference of the IEEE Engineering in Medicine and Biology Society IEEE Engineering in Medicine and Biology Society Conference 2011;**2011**:8219-22 doi: 10.1109/iembs.2011.6092027[published Online First: Epub Date]|.

76. Schweitzer EJ. Reconciliation of the cloud computing model with US federal electronic health record regulations. Journal of the American Medical Informatics Association : JAMIA 2012;**19**(2):161-5 doi: 10.1136/amiajnl-2011-000162[published Online First: Epub Date]|.

77. Shen CP, Chen WH, Chen JM, et al. Bio-signal analysis system design with support vector machines based on cloud computing service architecture. Conference proceedings : Annual International Conference of the IEEE Engineering in Medicine and Biology Society IEEE Engineering in Medicine and Biology Society Conference 2010;**2010**:1421-4 doi: 10.1109/iembs.2010.5626713[published Online First: Epub Date]|.

78. Shen CP, Jigjidsuren C, Dorjgochoo S, et al. A data-mining framework for transnational healthcare system. Journal of medical systems 2012;**36**(4):2565-75 doi: 10.1007/s10916-011-9729-7[published Online First: Epub Date]|.

79. Shih FJ, Fan YW, Chiu CM, et al. The dilemma of "to be or not to be": developing electronically e-health & cloud computing documents for overseas transplant patients from Taiwan organ transplant health professionals' perspective. Transplantation proceedings 2012;**44**(4):835-8 doi: 10.1016/j.transproceed.2012.02.001[published Online First: Epub Date]|.

80. Siddiqui Z, Abdullah AH, Khan MK, et al. Smart environment as a service: three factor cloud based user authentication for telecare medical information system. Journal of medical systems 2014;**38**(1):9997 doi: 10.1007/s10916-013-9997-5[published Online First: Epub Date]|.

81. Silva LA, Costa C, Oliveira JL. A PACS archive architecture supported on cloud services. International journal of computer assisted radiology and surgery 2012;**7**(3):349-58 doi: 10.1007/s11548-011-0625-x[published Online First: Epub Date]|.

82. Silva LA, Costa C, Oliveira JL. DICOM relay over the cloud. International journal of computer assisted radiology and surgery 2013;**8**(3):323-33 doi: 10.1007/s11548-012-0785-3[published Online First: Epub Date]|.

83. Singh S, Bansal M, Maheshwari P, et al. American Society of Echocardiography: Remote Echocardiography with Web-Based Assessments for Referrals at a Distance (ASE-REWARD) Study. Journal of the American Society of Echocardiography : official publication of the American Society of Echocardiography 2013;**26**(3):221-33 doi: 10.1016/j.echo.2012.12.012[published Online First: Epub Date]|.

84. Sofka M, Ralovich K, Zhang J, et al. Progressive data transmission for anatomical landmark detection in a cloud. Methods of information in medicine 2012;**51**(3):268-78 doi: 10.3414/me11-02-0017[published Online First: Epub Date]|.

85. Stoicu-Tivadar L, Stoicu-Tivadar V, Berian D, et al. eduCRATE--a Virtual Hospital architecture. Studies in health technology and informatics 2014;**205**:803-7 doi: 10.1371/journal.pone.0105297[published Online First: Epub Date]|.

86. Su CJ, Chiang CY. IAServ: an intelligent home care web services platform in a cloud for aging-in-place. International journal of environmental research and public health 2013;**10**(11):6106-30 doi: 10.3390/ijerph10116106[published Online First: Epub Date]|.

87. Takeuchi H, Mayuzumi Y, Kodama N, et al. Personal healthcare system using cloud computing. Studies in health technology and informatics. 2013/08/08 ed, 2013:936.

88. Tan CH, Teh YW. Synthetic hardware performance analysis in virtualized cloud environment for healthcare organization. Journal of medical systems 2013;**37**(4):9950 doi: 10.1007/s10916-013-9950-7[published Online First: Epub Date]|.

89. Tseng KC, Wu CC. An expert fitness diagnosis system based on elastic cloud computing. TheScientificWorldJournal 2014;**2014**:981207 doi: 10.1155/2014/981207[published Online First: Epub Date]|.

90. Van Gorp P, Comuzzi M. Lifelong personal health data and application software via virtual machines in the cloud. IEEE journal of biomedical and health informatics 2014;**18**(1):36-45 doi: 10.1109/jbhi.2013.2257821[published Online First: Epub Date]|.

91. Vida MM, Lupse OS, Stoicu-Tivadar L, et al. Flexible solution for interoperable cloud healthcare systems. Studies in health technology and informatics 2012;**180**:280-4

92. Vilaplana J, Solsona F, Abella, et al. The cloud paradigm applied to e-Health. BMC medical informatics and decision making 2013;**13**:35 doi: 10.1186/1472-6947-13-35[published Online First: Epub Date]|.

93. Wang H, Wu Q, Qin B, et al. FRR: fair remote retrieval of outsourced private medical records in electronic health networks. Journal of biomedical informatics 2014;**50**:226-33 doi: 10.1016/j.jbi.2014.02.008[published Online First: Epub Date]|.

94. Wang X, Gui Q, Liu B, et al. Enabling smart personalized healthcare: a hybrid mobile-cloud approach for ECG telemonitoring. IEEE journal of biomedical and health informatics 2014;**18**(3):739-45 doi: 10.1109/jbhi.2013.2286157[published Online First: Epub Date]|.

95. Xia H, Asif I, Zhao X. Cloud-ECG for real time ECG monitoring and analysis. Computer methods and programs in biomedicine 2013;**110**(3):253-9 doi: 10.1016/j.cmpb.2012.11.008[published Online First: Epub Date]|.

96. Xu R, Mei G, Zhang G, et al. TPM: cloud-based tele-PTSD monitor using multi-dimensional information. Studies in health technology and informatics 2013;**184**:471-7

97. Yao Q, Han X, Ma XK, et al. Cloud-based hospital information system as a service for grassroots healthcare institutions. TheScientificWorldJournal 2014;**38**(9):104 doi: 10.1155/2014/89436210.1007/s10916-014-0104-3[published Online First: Epub Date]|.

98. Yoo S, Kim S, Kim T, et al. Economic analysis of cloud-based desktop virtualization implementation at a hospital. BMC medical informatics and decision making 2012;**12**:119 doi: 10.1186/1472-6947-12-119[published Online First: Epub Date]|.

99. Yoo S, Kim S, Kim T, et al. Implementation Issues of Virtual Desktop Infrastructure and Its Case Study for a Physician's Round at Seoul National University Bundang Hospital. Healthcare informatics research 2012;**18**(4):259-565 doi: 10.4258/hir.2012.18.4.259[published Online First: Epub Date]|.

100. Yoshida H, Wu Y, Cai W, et al. Scalable, high-performance 3D imaging software platform: system architecture and application to virtual colonoscopy. Conference proceedings : Annual International Conference of the IEEE Engineering in Medicine and Biology Society IEEE Engineering in Medicine and Biology Society Conference 2012;**2012**:3994-7 doi: 10.1109/embc.2012.6346842[published Online First: Epub Date]|.

101. Yu HJ, Lai HS, Chen KH, et al. A sharable cloud-based pancreaticoduodenectomy collaborative database for physicians: Emphasis on security and clinical rule supporting. Computer methods and programs in biomedicine 2013;**111**(2):488-97 doi: 10.1016/j.cmpb.2013.04.019[published Online First: Epub Date]|.

102. Zao JK, Gan TT, You CK, et al. Pervasive brain monitoring and data sharing based on multi-tier distributed computing and linked data technology. Frontiers in human neuroscience 2014;**8**:370 doi: 10.3389/fnhum.2014.00370[published Online First: Epub Date]|.

Authors	Article type	Imple-menta-tion status	Domain	Description of cloud computing use	Objectives of the paper/the project	Users	Proprietary / commercially development	Why is/should cloud computing (be) used?	Concerns	Handling with concerns
Abbas, Khan [1]	Journal Paper	Theoretical work (no project)	Hospital Management	Store and access health records	Review on privacy in cloud environments	Patients, physicians, all	X	Storage, availability	Security concerns: violation of confidentiality and of data integrity, access to data by unauthorized persons	X
Ahnn, Potkonjak [2]	Journal Paper	Theoretical work (no project)	Public Health	X	To model cloud systems with focus on energy saving	X	X	X	Energy consumption	X
Ahuja et al. [3]	Journal Paper	Theoretical work (no project)	Other domain	X	To review of current use of cloud computing in healthcare (no systematic or scoping	X	X	X	X	X

					review)					
Almashaqbeh et al. [4]	Journal Paper	Prototype	Telehealth	Store and process	Use cloud as backend for body area networks	Patients, physicians		Broad network access, energy-efficiency	Security, dependence on cloud provider; lack of communication standards (cloud-mobile systems), loss of data	Multi-hop communication for the local cloud
Al-Zaiti et al. [5]	Journal Paper	Theoretical work (no project)	Telehealth	Store and provide	Review the state of wireless ECG transmission: collect data prior to admission to prepare rescue teams	Physicians	X	Broad network access	X	X
Amland, Hahn-Cover [6]	Journal Paper	Successful implementation	Secondary use of clinical data	Datamining in a retrospective study to learn about sepsis	Evaluate the results for early sepsis recognition using St. John Sepsis Agent (a Cerner Millenium	Physicians	X	Resource pooling	X	X

					Plugin)					
Avila-Garcia et al. [7]	Con-ference Paper	Conceptual work	Medical Imaging	Cloud-based framework for colorectal cancer imaging analysis and research for clinical use	To propose a cloud-based framework for colorectal cancer imaging analysis and research for clinical use	Clinical researchers	X	X	X	X
Bahga, Madisetti [8]	Journal Paper	Prototype	Hospital management	Store, process, provide	Bring a HIS (VistA) into the cloud as SaaS	Patients, physicians	Proprietary development	On-demand; measured service	Lack of data interoperability standards; data security	HIPAA conform approach based on identity management
Balkman, Loehfelm[9]	Journal Paper	Prototype	Others	Store, provide	Radiologists provide images useful for educating others	Physicians, students	X	Rapid elasticity, broad network access	X	X
Botts et al. [10]	Journal Paper	Success-ful implementation	Public Health	Cloud-based platform to provide health IT to underserved populations	To describe the platform	Patients, physicians	Commercial development	Broad network access	Lack of confidence of patients	X
Chang et al.[11]	Journal	Success-ful	Therapy	iSMART, cloud computing web	iSMART should	Clinical	Proprietary	X	X	X

	Paper	impleme ntation		server for analysis of Traditional Chinese Medicine studies and new drug design	become a web system that bridges traditional Chinese medicine and computer-based drug design	researchers	development			
Chard et al.[12]	Con-ference Paper	Proto-type	Seconda ry use of data	SaaS in a cloud for NLP	To propose to use CC to allow interactive real-time NLP applications , and outline exemplary user scenarios for their Smntx medical NLP architecture	Clinical researchers	Proprietary development	Rapid elasticity	Access to data by unauthorized persons, violation of confidentiality and integrity of data	HIPAA compliant environment to host services, authentication and authorization mechanisms, secure communication channels
Chen et al. [13]	Journal Paper	Theoretic al work (no project)	Teleheal th	PHR access control scheme under cloud computing environment	To propose to use clouds as infrastructu re for personal health records that can be	Medical professional s, patients	X	Reduced cost, dynamic scalability of resources, enhanced flexibility, elimination	Access to data by unauthorized persons, violation of confidentiality and integrity of data	PHR access control scheme, PHR encryption

					under patients' control			of device limitation		
Chen et al.[14]	Journal Paper	Concept-ual work	Tele-health	Infrastructure for EHRs	To discuss "security and privacy issues" of cloud-based EHRs. The authors postulate clouds as possibility for patients to control their data.	Patients, physicians	X	Rapid elasticity	Access to data by unauthorized persons, in emergency case access to encrypted data difficult	Electronic signatures, keys, access licenses; in emergency: emergency access key
Cheng et al. [15]	Conf-erence Paper	Proto-type	Tele-health	Cancer reporting and monitoring system caREMOTE	To improve doctor-patient interaction for domestic cancer care	Patients, physicians	X	X	Confidentiality of data	Encryption of data
Christoph et al. [16]	Journal Paper	Prototype	Secondary use	Processing of data (textmining)	Providing a privacy-aware computing infrastructure	IT departments	Proprietary development	X	Security concerns; violation of confidentiality and of data integrity, access to data by unauthorized	Deidentification of data; volatile processing in a technically secure cloud

									persons	
Cubo et al. [17]	Journal Paper	Prototyp e	Teleheal th	Store and process	Description of an architecture , modeling language and cost model for cloud-supported AAL (project DEEP)	Patients, physicians	X	Broad network access, measured service		
Dixon et al. [18]	Journal Paper	Proto-type	Therapy	Community cloud services to support clinical decision support	To present and evaluate of web-based clinical decision support services	physicians	Proprietary development	Broad network access	Violation of confidentiality and integrity of data, semantic interoperabilit y, need for monitoring	Incorporating only a limited data set of identifying information as defined by HIPAA
Dixon et al. [19]	Journal Paper	Prototyp e	Therapy	DSS as a service	Evaluate the DSS output with local rules	Physicians	Proprietary development	X	X	X
Doukas et al.[20]	Con-ference Paper	Proto-type	Medical imaging	Imaging applications on mobile devices	To evaluate cloud-based medical image and patient health	physicians	Proprietary development	On-demand self-service, broad network	Unauthorized access	Implementing advanced user authentication techniques as “future

					record sharing via a cloud-based infrastructu re provided by mobile application			access, resource pooling, rapid elasticity, measured service		research”
Doukas et al. [21]	Con-ference Paper	Proto-type	Other domain	Cloud-based image analysis of skin lesions	To detect skin cancer early using cloud-based data processing components	Patients	Proprietary development	X	X	X
Eriksson et al. [22]	Con-ference Paper	Proto-type	Public Health	Simulation architecture for simulating pandemic influenza outbreaks	To simulate pandemic influenza outbreaks using cloud resources to save money	Clinical researchers	Proprietary development	Rapid elasticity	Network capacity of the master node could become a bottleneck, violation of confidentiality and integrity of data	Industry-standard SSL encryption
Fekr et al. [23]	Journal Paper	Prototyp e	Others	process	Algorithms are too computing intensive tob e performed on wearables	Physicians	X	X	X	X

Ferenchick, Solomon [24]	Journal Paper	Successful implementation	Others	Web server	Digital questionnaire for evaluators of student performance	Educators	Proprietary development	On-demand self-service, broad network access	X	X
Fernandez-Cardenosa et al. [25]	Journal Paper	Concept-ual work	Tele-health	Infrastructure for mobile data access to realize e-health services	To estimate storage capacities and broadcast capacities for a hypothetic change to cloud-based EHRs for a "hospital" and a "primary care center" and calculated on basis of amazon pricelists	Physicians, other medical staff	X	On-demand self-service, resource pooling, rapid elasticity	Violation of confidentiality and integrity of data	Inform patients of migration of their data to the cloud
Fong, Chung [26]	Journal Paper	Prototype	Telehealth	Store and provide	Collect wireless ECG data and provide access through a	Physicians	X	Broad network access	X	X

					web portal					
Fujita et al. [27]	Con-ference Paper	Proto-type	Tele-health	Electrocardiography (ECG) system, share ECG inside and outside the hospital	To propose that cloud-based data sharing could be solution for emergency service for cardiology	Physicians	Commercial development	X	X	X
Garcia-Penalvo, Cruz-Benito [28]	Journal Paper	Prototype	Others	Virtual lab	Analysis of data gathered from a virtual pharma lab built in second life to train QM activities	Research laboratories, educators	X	X	X	X

Gerard et al. [29]	Journal Paper	Theoretical work (no project)	Medical Imaging	Cloud-based PACS	Explore advantages of cloud computing for radiologists	Patients, physicians (radiologists)	X	Broad network access, resource pooling	Security concerns, migration into cloud complex, lack of organizational best practices, data access	X
Glasberg et al. [30]	Journal Paper	Theoretical work (no project)	Hospital management	Outsource HIT	Risk management in hospitals	X	X	X	Security concerns; violation of confidentiality and of data integrity, access to data by unauthorized persons, violation of data protection laws	X

Haufe [31]	Journal Paper	Prototyp e	Hospital manage ment	Outsource HIT	Proposal of a security managemen t framework for outsources HIT	Hospital managemen t	X	Rapid elasticity, measured service	Loss of data, violation of confidentiality and integrity of data, only incomplete data delation possible, violation of data protection laws, access to data by unauthorized persosn	Providing a security management framework that supports requirements definition and auditing
He et al. [32]	Journal Paper	Proto-type	Public Health	Health cloud platform to provide self-monitoring of health	To enable "pervasive, economical, and on-demand services" and "flexibility and scalability" serve citizens to health monitor themselves via Internet and support physicians	Physicians, patients	Proprietary development	Broad network access	Loss of data (“data may possibly be lost by accident, e.g. power failure”)	Maintain enough replicas

Hiden et al. [33]	Journal Paper	Proto-type	Tele-health	Infrastructure for scientists to store, analyze and share data from MOVEeCloud project	To make it easier for scientists to store, share and analyze their data, and for developers to create new scientific services and applications	Clinical researchers	Proprietary development	On-demand self-service, rapid elasticity	Violation of confidentiality and integrity of data, access to data y unauthorized persons	Access control lists (ACL), user authentication
Hussain et al. [34]	Journal Paper	Prototyp e	Teleheal th	Store and process	Collect data via mobile devices and process it in the cloud	X	X	Rapid elasticity, broad network access	X	X
Hsieh, Hsu [35]	Journal Paper	Proto-type	Tele-health	Visualization of ECG data utilizing a cloud service	To present a cloud-based telemedicin e system for ECG diagnosis based on standardize d data formats, to support inter-hospital and pre-hospital consultation , and to	Physicians	Proprietary development	Broad network access, on-demand self-service	Loss of data, violation of confidentiality and integrity of data	Authentication for the use of Web roles, data encryption

					enhance interoperability					
Jalali et al. [36]	Journal Paper	Concept-ual work	Public Health	Public health reporting and information exchange	To evaluate how a secure virtual private cloud (VPC) solution could facilitate the implementation of the Smarter Public Health Prevention System	Patients, physicians	X	Broad network access, rapid elasticity	Unauthorized access	Utilization of secure virtual private clouds
Ji, Ganchev [37]	Journal Paper	Prototype	Telehealth	Store, process, provide	Body Area Network collects data, the cloud processes and forwards them	patients	X	X	X	X
Kagadis et al. [38]	Journal Paper	Theoretical work (no	Medical Imaging	X	To describe Vision20/20 for CC in imaging	X	X	X	Data protection and privacy, loss of data	Service level agreements with the provider (e.g.

		project)			(cloud-based PACS)				control dependence on cloud provider	concerning encryption and safety policies)
Kharat et al. [39]	Journal Paper	Theoretical work (no project)	Medical Imaging	X	To explain cloud computing to radiologists	Physicians	X	On-demand self-service, broad network access, resource pooling, rapid elasticity, measured service	Data security and privacy, reliability, availability	Encryption, audit trails, service level agreements
Klein [40]	Journal Paper	Theoretical work (no project)	Other domain	X	To review privacy rulings in the light of (cloud-based) services (such as Dropbox)	X	X	Broad network access, on-demand self-service	Violation of data protection laws, confidentiality, privacy	X
Kondoh et al. [41]	Con-ference Paper	Proto-type	Tele-health	Remote access to electronic patient record systems and PACS	To provide server based computing system as infrastructure of the	Physicians	X	X	X	X

					total integrated PACS and EPR					
Koufi et al. [42]	Con-ference Paper	Proto-type	Tele-health	Cloud-based emergency system integrating personal health records	To provide physicians with easy and immediate access to patient data from anywhere and via almost any computing device while containing costs	Physicians	Proprietary development	Broad network access	Violation of confidentiality and integrity of data	X
Kuo [43]	Journal Paper	Theoretic al work (no project)	Other domain	X	To discuss the concept of cloud computing, its current applications in health care, the challenges and opportuniti es, and how to implement strategic	X	X	On-demand self-service, broad network access, resource pooling, rapid elasticity, measured service	Security and privacy concerns	X

					planning when the organization has decided to move to the new model of service					
Kuo et al. [44]	Con-ference Paper	Theoretical work (no project)	Other domain	X	To conduct a SWOT-Analysis of cloud computing in healthcare	X	X	On-demand self-service, broad network access, resource pooling, rapid elasticity, measured service	Security and privacy concerns, insufficient evidence of success, reliability, network traffic	X
Lai et al. [45]	Journal Paper	Prototype	Telehealth	Store and process	Use cloud as backend to reconstruct 3D postures based on BANs (collaborative framework)	Patients, medical care personnel	X	Rapid elasticity, broad network access	Data transfer costly (battery life of sensors)	X
Langer [46]	Journal Paper	Concept-ual work	Medical Imaging	X	To conceptualize cloud	Clinical reserachers,	X	Rapid elasticity, measured	Security, privacy	HIPAA-compliant

					computing for (research) data storage in imaging research	physicians		service		anonymization
Lenert, Sundwall [47]	Journal Paper	Theoretical work (no project)	Public Health	X	To show cloud computing as a solution to the crisis of fulfilling regulatory demands	Clinical researchers	X	Resource pooling, broad network access	Lack of harmonized national legislation, security, privacy, intrusion	X
Low, Hsueh [48]	Journal Paper	Theoretical work (no project)	Hospital manage-ment	X	To provide decision criteria for choosing (cloud) outsourcing partners for hospital information systems	X	X	X	X	X
Lupse et al. [49]	Journal Paper	Theoretical work (no project)	Hospital manage-ment	X	To discuss Cloud computing and interoperability for exchange of	X	X	Broad network access, rapid elasticity	Lack of technical, legal, economic and security details, ownership of	CDA/HL7 should be used to standardize data exchange

					data between department s				data	
Maratt et al. [50]	Journal Paper	Proto-type	Medical Imaging	Cloud-based digital templating system for planning total hip arthroplasty	To use SaaS-Tools for planning total hip arthroplasty	Physicians	Proprietary development	Measured service	Security, privacy	HIPAA-complaint local encryption, service level agreement with provider
Meng et al. [51]	Journal Paper	Proto-type	Medical Imaging	MapReduce algorithms for CT-scan reconstruction	To show utility of MapReduce algorithms for CT-scan reconstructi on as example for solving larg-scale medical physics problems	Programmer s	X	Rapid elasticity	Network traffic	X
Miras et al. [52]	Journal Paper	Proto-type	Therapy	Monte Carlo simulation in the cloud to support radiotherapy planning	To describe Monte Carlo simulation in the cloud to support radiotherap y planning	Programmer s	Proprietary development	Rapid elasticity, broad network access, measured service	SaaS tend to be rigid/no customization , need programming skills, network traffic	X
Mirza, El-Masri [53]	Con-ference	Concept-	Tele-	Cloud system for unification and	To enable sharing and	Physicians	X	Broad network	Security,	Privacy is ensured by

	Paper	ual work	health	integration of EHR to share patient information among healthcare providers	exchange of patient data between hospitals and healthcare providers			access	privacy	patient authorizing mechanism
Morton et al. [54]	Co-nference Paper	Proto-type	Tele-health	Location-tracking sensor with back-end cloud data processing	To present a prototype integrating a wearable location-tracking sensor with back-end cloud-based data processing, thereby enabling real-time tracking and analysis of a large number of people simultaneou sly.	Programmer s	Proprietary development	Rapid elasticity	X	X
Na et al. [55]	Journal Paper	Concept-ual work	Therapy	Web-based treatment planning system	To propose a strategy developing a web-based therapy	X	Commercial development	On-demand self-service, rapid	Cost, security concerns, data privacy, network can become congested	De-identified clinical cases encrypted by the 256-bit AES algorithm (NIST-FIPS standard)

					planning system in cloud environmen t			elasticity	when multiple data packages are concurrently transferred across the network	before transmission, transmission through secure socket layer encrypted endpoints, private and public keys for unique identification; HIPAA compliance
Nagata et al. [56]	Journal Paper	Success-ful imple-mentatio n	Other domain	Cloud-based EHR for disaster mitigation in Fukushima	To report on using a cloud-based EHR during disaster mitigation in Fukushima	Physicians	Proprietary development	Broad network access, rapid elasticity	X	Virtual private networks
Nkosi, Mekuria [57]	Con-ference Paper	Concept-ual work	Other domain	Cloud computing protocol management system that provides multimedia sensor signal processing and security as a service to mobile devices	To improve utilization of ubiquitous mobile devices for societal services and promote health service delivery to marginalize d rural	X	X	X	Security, privacy concerns	Security updates over the cloud → security as a service

					communitie s					
Parsons et al. [58]	Journal Paper	Prototyp e	Therapy	Model for Monte Carlo simulation of radiation dose	Demonstrati on and validation of VirtuaLinac with clinical photon beams	Clinical researchers, physicians	Commercially development	X	X	X
Papakonstantinou et al. [59]	Con-ference Paper	Proto-type	Other domain	Cloud system to support training in healthcare process management	To provide a prototype system (semantic wiki) of knowledge acquisition	Medical professional s	X	Broad network access, rapid elasticity, measured service	Unauthorized access	Virtual private networks
Patel [60]	Journal Paper	Theoretic al work (no project)	Medical Imaging	X	To investigate applications of cloud computing in a radiology department	X	X	Rapid elasticity, resource pooling, measured service	Data confidentiality , data integrity	Secure location of provider, authentication, secure socket layer
Piette et al. [61]	Journal Paper	Success-ful imple-men-tation	Public Health	Self-management calls plus home blood pressure monitoring	To evaluate automated self-managemen t calls plus home blood pressure monitoring	Patients	Proprietary development	Broad network access	X	X

					as strategy for improving systolic BPs					
Piette et al. [62]	Journal Paper	Success-ful imple-men-tation	Public Health	Self-management calls with information for patients in Honduras how to manage their diabetes	To report on providing 85 diabetes patients in Honduras with weekly interactive voice response calls for six weeks with recorded information in Spain about how to manage their disease	Patients	Proprietary development	Broad network access	X	X
Poole et al. [63]	Journal Paper	Proto-type	Therapy	Radiotherapy Monte Carlo simulation, application GEANT4	To run the application GEANT4 on a cloud infrastructu re	Physicians	Proprietary development	Rapid elasticity	X	X
Poulymenopoulou et al. [64]	Journal Paper	Proto-type	Tele-health	Networking between emergency departments	To develop an integrated computer support to	Physicians	Proprietary development	Broad network access	Access to data by unauthorized persons, violation of	Context-aware authorizations, two servers enforce local and global

				and ambulance	emergency care processes by evolving and cross-linking institutional healthcare systems.				data protection laws	security policies
Price et al. [65]	Journal Paper	Proto-type	Public Health	Epidemiology: to run the application SaTScan for detection of cluster characteristics in a cloud environment	To run the computer intensive application SaTScan for detection of cluster characteristics in a cloud environment	Public Health Departments, epidemiologists	Proprietary development	Rapid elasticity, on-demand self-service	X	X
Qi et al. [66]	Journal Paper	Proto-type	Medical Imaging	Image texture analysis, transformation of pathology images	To investigate the robustness of a range of image texture features using hematoxylin stained breast	Physicians	Proprietary development	Rapid elasticity, on-demand self-service	X	X

					tissue microarray slides					
Rajkumar, Sriman Narayana Iyengar [67]	Journal Paper	Prototyp e	Teleheal th	Data centers in cloud for P2P network for processes in emergency healthcare	Enable more efficient treatment of emergency patients	clinical researchers, medical professional	Proprietary development	Resource pooling	X	X
Rao et al. [68]	Con-ference Paper	Proto-type	Tele-health	Pervasive cloud initiative Dhatri to leverage the power of cloud computing in wireless technologies	To describe Dhatri	Physicians, patients	Commercial development	Broad network access	Violation of confidentiality and integrity of data, access to data by unauthorized persons	Advanced Encryption Standards (AES) algorithms to encrypt datasets, role-based access
Ratnam et al. [69]	Journal Paper	Concept ual work	Hospital manage ment	Cloud computing platform to host the Malaysian national healthcare information exchange	Find, if cloud computing could is able and efficient when hosting the national healthcare information exchange	Government , policy	X	X	X	X
Rea et al. [70]	Journal Paper	Proto-type	Secon-dary use	Text mining cloud and data exchange via	To create a platform for NLP and	Clinical researchers,	X	X	Data security and privacy	X

			of data	cloud	data sharing between hospitals	physicians			concerns	
Regola, Chawla [71]	Journal Paper	Proto-type	Secon-dary use of data	Secure storage of health data in a private cloud for research purposes	To discuss and demonstrate how to set up data transfer and storage in a cloud in HIPAA complying environment	Clinical researchers	Proprietary development	Rapid elasticity	violation of confidentiality and integrity of data, violation of data protection laws, access to data by unauthorized persons	administrative policies, physical safeguards for facility access and workstation security, authentication / authorization, anonymized patient data; HIPAA compliance
Ribeiro et al. [72]	Journal Paper	Concept-ual work	Tele-health	Networking between healthcare institutions	To present a framework for define manage and test IHE (integrating healthcare enterprise) affinity domains	X	Proprietary development	Broad network access, resource pooling	Violation of data protection laws	Profiles, audit trails, node authentication
Rodrigues et al. [73]	Journal Paper	Theoretical work (no project)	Tele-health	X	To analyze how healthcare providers and clinical centers can guarantee	Physicians	X	Rapid elasticity	Privacy, security, confidentiality of patients' data	Cloud clients should be well informed before moving data to the cloud, e.g. about security

					the security, privacy, and confidentiality of their patients' data					mechanisms installed on the provider's servers. Cloud clients should demand total transparency from the cloud provider.
Rolim et al. [74]	Conference Paper	Conceptual work	Telehealth	Cloud-based system to automate the process of collecting patient's vital data via a sensor network, data storage, processing, and distribution in a cloud	To propose a solution to automate the process of collecting patients' vital data by using sensors attached to existing medical equipments that are inter-connected to exchange service	Physicians	X	Resource pooling	Violation of confidentiality and integrity of data	X
Rostrom, Teng [75]	Conference Paper	Prototype	Medical Imaging	PACS storage	To demonstrate secure connection of local client to cloud	Physicians	Proprietary development	rapid elasticity, more robust cost-effective business	access to data by unauthorized persons, violation of confidentiality and integrity	trusted certificate authority (CA) which creates a certificate for both the cloud application and

					storage			continuity planning, reduced manageme nt decisions concerning infrastruct ure, scalability, increased resiliency and cost reductions	of data, violation of data protection laws	the client, encryption, secure communication policy; HIPAA compliance
Schweitzer [76]	Journal Paper	Theoretic al work (no project)	Tele-health	Cloud-based EHRs	To discuss security issues of cloud based EHRs	X	X	on-demand self-service, broad network access, resource pooling, rapid elasticity, measured service	Security and privacy concerns, loss of data, data cannot be deleted completely, hidden costs (e.g. legal fees for writing contracts and defending disputes with the cloud provider, salaries of security staff to monitor the provider's performance),	HIPAA compliance; Economic analysis, risk analyses; implementation of policies and procedures to address vulnerabilities; assign responsibility to a security officer; screen and educate its work force; limit workforce access to personalized health

									concerns about maturity of the service	information, development of incident response plans; protection and limitation of access to servers; storage media, and workstations; management of user identity; encryption of data, monitoring of system activity; business associate contract
Shen et al. [77]	Con-ference Paper	Success-ful imple-men-tation	Other domain	Bio-signal analysis (e.g. EEG, ECG data)	To demonstrat e web based service oriented system for bio signal analysis	Physicians	Proprietary development	X	X	X
Shen et al. [78]	Journal Paper	Proto-type	Secon-dary use of data	Data mining to learn clinical pathways from order sets documented in the EHR	To describe generic standards-based services that can be transferred	Clinical researchers	Proprietary development	X	Data security, data privacy	authorization, authentication, using a generated session key, deidentification

					as virtual machines to other hospitals					
Shih et al. [79]	Co-nference Paper	Theoretical work (no project)	Tele-health	exchange of medical documents between Taiwan and China	To develop mutually accessible e-health documents and cloud computing for overseas organ transplants	Physicians	X	Resource pooling	X	X
Siddiqui et al. [80]	Journal Paper	Prototype	Public Health	Telecare Medical Information System with medical services, cloud to enable use on smartphone	Proposal of Three Factor remote user authentication protocol utilizing a smartphone capability	Patients, Physicians	Hybrid Cloud	X	Security, violation of confidentiality	X
Silva et al. [81]	Journal Paper	Success-ful imple-men-tation	Medical Imaging	PACS storage, cloud-based PACS as a service	To propose cloud based PACS as a service	Physicians	Proprietary development	resource pooling, on-demand self-service	Dependence on cloud provider, absence of interface normalization, Internet latency, loss of data, violation of	authorization, encryption, storage of all identifiable data in separate entity called Master Index

									confidentiality and integrity of data, access to data by unauthorized persons	
Silva et al. [82]	Journal Paper	Success-ful imple-men-tation	Medical Imaging	DICOM-compliant bridge for extending and sharing DICOM services across healthcare institutions	To develop a DICOM-compliant bridge	Physicians	Proprietary development	Resource pooling	X	X
Singh et al. [83]	Journal Paper	Success-ful imple-mentatio n	Tele-health	Web-based tele-consultation of remote physicians to interpret cardio sonographic images	To test feasibility of performing focused echocardiog raphic studies with long-distance web-based assessments of recorded images for facilitating care of patients with cardiovascul	Physicians	Proprietary development	Broad network access	violation of confidentiality and integrity of data	multiple integrity checks during the transmission process + standard Secure Sockets Layer (Transport Layer Security) encryption

					ar disease					
Sofka et al. [84]	Journal Paper	Proto-type	Medical Imaging	progressive data transmission for remote image analysis	To enable efficient transport of large image files between PACS and Image analysis server	Physicians	Commercial development	Broad network access, resource pooling	access by unauthorized persons	Provide secure access
Stoicu-Tivadar et al. [85]	Journal Paper	Prototyp e	Others	Information system that provides training for medical students to treat patients in second life environment	Enable medical students' training with simulated reality	Ongoing physicians	Commercial development	X	X	X
Su, Chiang [86]	Journal Paper	Prototyp e	Public Health	IAServe (Intelligent Aging-in-Place Home care web services platform), Provision of applications like reminders, weather information	Provision of healthcare services for elderly people to enable them to stay at home	Patients, healthy, elderly persons at home	X	Economic, scalable and ubiquitousl y accessible service provision	X	X
Takeuchi et al. [87]	Con-ference	Proto-	Public	Personal health and lifestyle	To provide users with	Patients	X	X	X	X

	Paper	type	Health	data stored in a cloud through mobile devices. Automatic extraction of personally useful information using data mining technologies	information and advices on their health managemen t and lifestyle.					
Tan, Teh [88]	Journal Paper	Proto-type	Hospital manage-ment	monitoring hardware performance and consistency, envisage the mechanism to construct stress-testing scenario	To enable performanc e evaluation of the cloud resources (esp. Databases) without real patient data	Hospital IT staff	Proprietary development	Rapid elasticity	hardware failure, violation of data protection laws, access to data by unauthorized persons	multi-layer authentication, encrypted data, multi-copy of data backup
Tseng, Wu [89]	Journal Paper	Prototyp e	Public Health	Expert system of a cloud infrastructure to help older people diagnose their health status	Enable customized diagnosis	Elderly people	X	Automatic ally allocate computati onal resources to maximize the quality of service to the client	X	X

								while saving computing resources		
Van Gorp, Comuzzi [90]	Journal Paper	Prototype	Public Health	Cloud-based personal health record	MyPHRMachines should remain the lifelong property of patients who should be able to show them to selected caregivers	Patients	X	Sharing, saving medical data	X	X
Vida et al. [91]	Conference Paper	Conceptual work	Hospital management	data exchange between two systems via HL7	To present a Web Custom Control that shows the database tables and fields of available source systems and thus facilitates configuration of the local system in order to prepare it	Hospital IT staff	X	Rapid elasticity, measured service	violation of data protection laws, access to data by unauthorized persons	HTTPS and cryptographic algorithms

					for communication with other systems					
Vilaplana et al. [92]	Journal Paper	Theoretical work (no project)	Tele-health	automatic resource allocation for eHealth cloud applications, improve queuing performance	To model performance to enable automatic resource allocation in order to deploy a platform to support the computing needs of many hospitals, with different clinical departments	X	X	rapid elasticity, on-demand self-service, measured services	X	X
Wang et al. [93]	Journal Paper	Theoretical work (no project)	Telehealth	Cloud providers often not trustworthy	Retrieve encrypted medical records outsourced into a cloud	Hospital management	X	X	X	X
Wang et al. [94]	Journal Paper	Conceptual work	Telehealth	Cloud to store data from personal health	Provide solution to renovate	Patients, healthy	X	Computing and storing	X	X

				sensors and perform processing tasks	and promote the use of mobile devices in healthcare with cloud computing	persons		resources		
Xia et al. [95]	Journal Paper	Proto-type	Tele-health	home ECG monitoring over mobile devices	To provide cloud-based system for real time ECG monitoring and remote diagnostics	Patients, physicians	Proprietary development	on-demand self-service, broad network access, resource pooling, rapid elasticity, measured service	access to data by unauthorized persons	HTTPS
Xu et al. [96]	Con-ference Paper	Proto-type	Public Health	Cloud-based stress disorder screening and monitoring tool	To present an automated cloud based tele-stress disorder monitor system	Patients	Proprietary development	X	X	X
Yao et al. [97]	Journal Paper	Prototyp e	Hospital manage ment	Pooling of computing resources and health software	Small healthcare facilities should get	Hospital managemen t	Proprietary development	Rapid service deploymen t,	X	X

				in clouds to share between large hospitals and small healthcare facilities in China	resources from larger hospitals to save costs			ubiquitous medical services, simplified and efficient manageme nt of HIT resources		
Yoo et al. [98]	Journal Paper	Theoretic al work (no project)	Hospital manage-ment	virtualization of hospital IT, estimation of costs	To evaluate economic validity of investing in virtualizatio n at a hospital	X	X	X	X	X
Yoo et al. [99]	Journal Paper	Success-ful imple-me-ntation	Hospital manage-ment	virtualization of hospital IT	To evaluate physician and patient satisfaction after introduction of i-pad based hospital IT virtual machines	Physicians	Proprietary development	Rapid elasticity	access to data by unauthorized persons	user authorization, employ transmission protocols such as PCoIP
Yoshida et al. [100]	Con-ference Paper	Success-ful imple-men-tation	Medical imaging	virtual 3D colonoscopy	To present a software platform to support the developmen t of high-	Physicians	Proprietary development	Resource pooling	X	X

					performance 3D medical image processing applications with focus on fragmentation and distribution of tasks					
Yu et al. [101]	Journal Paper	Success-ful imple-men-tation	Tele-health	Cloud-based pancreaticoduodenectomy collaborative database	To build an international web accessible database of pancreaticoduodenectomy patients	Physicians	Proprietary development	Resource pooling, measured service	violation of data protection laws, violation of confidentiality and integrity of data	authentication, secure socket layer, encryption in database
Zao et al. [102]	Journal Paper	Prototype	Telehealth	Online EEG-BCI (brain-computer interface) systems using Fog and Cloud Computing	Making BCI systems truly wearable to make reliable real-time predication of users' cognitive states	Patients, healthy persons	Proprietary development	X	Security concerns	X
